# Drug shortages in China: a cross-sectional study

**DOI:** 10.1186/s12913-023-09295-w

**Published:** 2023-05-04

**Authors:** Yinyin Song, Jianchun Li, Fei Zhao, Pengfei Jin

**Affiliations:** 1 Department of Pharmacy, Beijing Hospital; National Center of Gerontology; Institute of Geriatric Medicine, Chinese Academy of Medical Sciences; Beijing Key Laboratory of Assessment of Clinical Drugs Risk and Individual Application (Beijing Hospital), No. 1 Dahua Road, Dongcheng district, Beijing, 100730 P.R. China; 2 Department of Pharmacy Administration and Clinical Pharmacy, School of Pharmaceutical Science, Peking University, Beijing, China

**Keywords:** China_1_, Drug shortages_2_, Essential medicines_3_, Medical insurance_4_, Volume-based purchasing_5_

## Abstract

**Background:**

Drug shortages significantly threaten public health and medical service provision worldwide. Research evidence on the complete picture of drug shortages is currently scant in China. This study aimed to provide a descriptive overview and a reference for alleviating of drug shortages in China.

**Methods:**

National and provincial lists of drug shortages issued in China from 2018 to 2021 were collected and summarized. The information on essential medicines, medical insurance drugs, emergency drugs, and volume-based purchasing drugs was then matched with a drug shortage list to analyse the characteristics, proportion and incidence of drug shortage on each list based on the analysis of information such as dosage form, shortage frequency, and Anatomical Therapeutic Chemical (ATC) classification of the drugs in shortage.

**Results:**

A total of 24 provinces issued drug shortages lists involving 408 drugs from 2018 to 2021. All 58 drugs in the national drug list were included on the provincial drug shortage list. Among all the drugs in shortage, the most significant shortage involved injections, accounting for 45.3% (185/408). Ninety-five drugs (23.3%) were in shortage 5 times (annual shortage > 1 time) or more in the provincial lists, and 199 drugs (48.8%) were on the shortage list only once. In terms of therapeutic property, nearly all categories of drugs had been reported in shortage, among which cardiovascular drugs, nervous system drugs, anti-tumor and immunomodulatory drugs, and blood and hematopoietic organ drugs accounted for more than 10%. There is no significant difference in drug shortage among economic regions. Comparing drugs in shortage and various lists, 81.9% (334/408), 51.0% (208/408) and 67.9% (277/408) fell on the National Medical Insurance Drug List, National Essential Medicines List, and WHO Model List of Essential Medicines, respectively, while the volume-based purchasing drugs accounted for 3.4% (14 drugs). The incidence of drug shortages on NEML, WHO Model List of Essential Medicines and medical insurance category A was significantly higher than that of medical insurance category B and volume-based purchasing drugs (P < 0.05). Of the Emergency Drugs List, 72.0% (36/50) also experienced shortages, significantly higher than all the above categories (P < 0.05).

**Conclusions:**

In China, drug shortages were severe and complicated. Drug shortages vary among economic regions but are not significant. In comparison, the national procurement pattern of volume-based drug purchasing may be conducive to alleviating the drug shortage problem. Collaboration of all partners was recommended to ensure the supply of clinically necessary drugs.

**Supplementary Information:**

The online version contains supplementary material available at 10.1186/s12913-023-09295-w.

## Background

The World Health Organization (WHO) has considered drug shortages a complex global challenge [1]. There is a lack of a standardized definition of drug shortage globally [2]. According to the WHO, a drug shortage is an insufficiency in the supply of medicines, health products, and vaccines are identified by the health system as essential to meet public health and patient needs [3]. It profoundly impacts patient safety, clinical outcomes, quality control, and healthcare facility management, posing complex challenges for healthcare providers [4]. Drug shortages have prevailed worldwide, affecting high-, middle-, and low-income countries. From 2012 to 2018, shortages of 3,530 pharmaceutical products, including 1,833 different active substances, were reported in France [5]. From 2014 to 2019, 209 drug shortages impacted medications on the WHO Model List of Essential Medicines for Children (EMLc), of which 77 (36.8%) remained unresolved by 2019 in the United States [4]. In 2021,there were 114 drug shortages in the United States [6]; the Dutch Medicines Evaluation Board received 3660 notifications of a lack of 1,835 medicines [7]. Although high-, middle- and low-income countries all deal with shortages, there have been few research studies about the prevalence of shortages in low- and middle-income countries [2]. Drug shortages substantially increased from 2020 to May 2021 due to the COVID-19 pandemic. There were 399 drugs classified as vital unavailable medicines in Colombia until May 2021 [8].

In China, drug shortage was defined as “the shortage of drugs that are clinically necessary, irreplaceable or not completely replaceable, and are in short supply or unstable supply in a certain period of time or in a certain region after being approved by the National Medical Products Administration (NMPA)” [9]. Identifying the characteristics of drug shortages can be beneficial to implementing targeted preventive measures. A qualitative study on drug shortages in Shaanxi Province was conducted in 2016, which indicated that drugs facing shortages were increasing, and essential medicines were mostly involved. The shortage of all 87 identified biologicals and chemicals was reported by tertiary and secondary hospitals in Shaanxi Province [10]. In 2017, a study to characterize the shortage of emergency drugs in China showed that the shortage was very severe. According to the survey, 90.7% of the respondents experienced drug shortages during the previous year. More than half of the physicians (65.7%) reported that drug shortages occurred at least once a month [11]. In 2018, an analysis of 614 hospitals in China showed that the average frequency of drug shortages in general hospitals was 56.1 times per year [12]. A national list of drug shortages involving 58 drugs was also released by National Health Commission of the People’s Republic of China on December 30th, 2020 [13].

The ongoing shortage of drugs has brought negative effects to health care, such as treatment discontinuation due to the unavailability of therapeutic drugs [11]. In particular, the shortage of some emergency medicines may delay treatment and directly threaten patients’ lives [14, 15]. Drug regulatory agencies in various countries attach more and more importance to drug shortages to solve the problem. The Chinese government implemented the national and provincial list management systems for drug shortages, requiring each province to collect, analyse and evaluate the information on drug shortages and formulate the provincial list of drug shortages and list of clinically essential but liable to shortage drugs (hereinafter referred to as “lists of drug shortages”).

Essential medicines were defined by WHO as a class of drugs satisfying the population’s priority healthcare needs and were defined in China as medicines that meet essential medical and health needs, with appropriate dosage forms, reasonable prices, guaranteed supply, and fair access to the public [16, 17]. To ensure public demands, the model and system of essential medicines have been established in China, and the “National Essential Medicine List” (NEML) was formulated through scientific and systematic selection [18]. The latest edition of the NEML was developed in 2018 [19]. The “National Medical Insurance Drug List” (NMIDL) is one of the carriers and manifestations of the basic Chinese medical insurance system. It is used to guide the use and reimbursement of drugs, reduce the burden of medical treatment, and protect the rights and interests of universal health insurance [20]. According to different payment standards, drugs on the list can be divided into category A and category B. Category A drugs are clinically necessary, widely used, and less expensive than other drugs with similar efficacy, while category B drugs refer to drugs that are also clinically necessary and effective but have a higher price than category A. Category A drugs can be reimbursed 100% through universal health insurance. In contrast, category B drugs can be reimbursed at a certain proportion, usually between 70% and 90% depending on local policies and specific drugs. The latest edition of the NMIDL was developed in 2021 [21]. Since 2019, the volume-based drug purchasing policy that aimed to further decrease drug prices by guaranteeing market shares was also implemented in China, and five batches of this kind of purchasing involving 218 drugs had been carried out successfully by June 2021 [22].

However, no comprehensive study was conducted to explore the full picture of drug shortages in China. This study was to collect the lists of drug shortages issued in China from 2018 to 2021 and summarize the characteristics, to provide references for the solution to drug shortages in China.

## Methods

### Data sources

This was a cross-sectional study of drug shortages in China from 2018 to 2021. We obtained drug shortage data from the official websites of the health commission and platforms of drug procurement and management in all 31 provincial administrative units of China’s mainland. The websites and platforms contain national and provincial lists of drug shortages. The registration approvals and manufacturer information of drugs and their active pharmaceutical ingredients (APIs) were collected on the NMPA website [23].

Six attributes were considered for the drugs: (1) the ATC classification of the WHO, (2) the 2018 edition of the NEML [16], (3) the 2021 edition of the WHO Model List of Essential Medicines[23], (4) the 2021 edition of the NMIDL [21], (5) the list of “Emergency (Rescue) Drugs Directly Online Procurement of Demonstration Drugs (Chemicals and Biological Products)” that involved 60 drugs and was released in 2015 (hereinafter referred to as the “Emergency Drug List” [EDL]) [24], and (6) the list of volume-based purchasing of five batches (hereinafter referred to as the “Volume-Based Purchasing List” [VBPL]).

### Data analysis

Summarize each province’s drug shortages from 2018 to 2021, and count their generic name, dosage form, province, WHO classification of ATC, and the cumulative number of shortages in four years. The list can be obtained by sorting the above drugs in descending order according to the frequency of shortages.

For drugs with a shortage frequency ≥ 5 times (an annual shortage > 1 time), compare them with the national list of drug shortages, and inquire about the manufacturer information of pharmaceutical drugs and APIs on the official website of the NMPA.

Compare the drug shortages list with the 2018 NEML, 2021 WHO Model List of Essential Medicines, 2021 NMIDL, 2015 EDL and VBPL, and determine the proportions of drug shortages in each list and drugs contained in other drug lists in shortages list. A chi-square test is used to check whether there is a difference in the ratio of drug shortage in each list.

The provinces are divided into four regions according to their geographical location and economic development level, and the provinces in each region are geographically adjacent and have a similar level of economic development. From high to low, the order of Gross Domestic Product is eastern, central, western and northeast [25]. ANOVA is used to determine whether there is a difference in the average annual number of drugs in shortages between economic regions.

IBM SPSS Statistics for Windows 26 is used for analysis with a significance level of 0.05.

### Patient and public involvement

All material in this study is derived from an official open access list and therefore does not require a research license or an ethics committee evaluation. Appropriate scientific practices and research ethics standards were followed throughout the research process.

## Results

A total of 408 drugs were involved in the issued provincial lists of drug shortages in China from 2018 to 2021 (Table S1 in Supplementary Material).

### Dosage form characteristics of drug shortages

Regarding dosage forms, oral dosage and injection accounted for 88.7% in total, with 43.4% (177/408) for oral and 45.3% (185/408) for injection, while topical (including eye drops, lotion, eye substance, cream, nasal drops, etc.) and others only accounted for 11.3%. Rank the list according to the shortage frequency. Injections accounted for 88.2% (45/51) of the top 25% and 76.5% (78/102) among the top 50%.

All top 5 drugs in shortages were injections: (1) methotrexate injection, (2) vitamin K1 injection, (3) posterior pituitary injection, (4) urokinase injection, and (5) protamine injection (Table 1).

### Ratio of drug shortages

In total, 48.8% (199/408) of the drugs were involved in the provincial lists once, and 15.7% (64/408) were involved twice (Figure S1 in Supplementary Material). The drugs with a shortage frequency of less than 5 (annual shortage frequency ≤ 1) account for about 3/4 of all the drugs in shortage; 93.1% (54/58) of the drugs on national drug shortage list appeared 5 times or more in provincial drug short orders (Table S1 in Supplementary Material).

All 95 drugs with a frequency ≥ 5 were produced by at least one manufacturer approved by the NMPA. There were 4 drugs (carbidopa/levodopa controlled / sustained-release tablet, clofazimine capsule, acetamide injection and estriol cream) that were produced by only one manufacturer, accounting for 4.2% (4/95), and 12 drugs were produced by fewer than three manufacturers. Most (73/95) were produced by a number of manufacturers ranging from 3 to 50, and 10 were even produced by more than 50 approved manufacturers. For APIs, 92.6% (88/95) were produced by at least one manufacturer. The frequency and manufacturer information for 95 drugs follow in Table 1.

**Table 1:** The manufacturer information for drugs with a frequency ≥ 5 in the provincial lists of drug shortages in China, 2018–2021

Ranking	Name	Dosage form	Frequency in the provincial shortage lists^*^	Number of API manufacturers	Number of drug manufacturers	Whether in the national list of drug shortages
1	Methotrexate	Injection	26	5	12	YES
2	Vitamin K	Injection	22	4	20	YES
3	Posterior pituitary	Injection	21	2	2	YES
4	Urokinase	Injection	21	2	8	YES
5	Protamine	Injection	21	5	29	YES
6	Atropine	Injection	20	3	84	YES
7	Lobeline	Injection	19	0	10	YES
8	Bleomycin	Injection	18	2	3	YES
9	Deslanoside	Injection	18	1	2	YES
10	Nikethamide	Injection	17	5	5	YES
11	Nitroglycerin	Injection	17	2	3	YES
12	Neostigmine	Injection	17	2	28	YES
13	Pyridostigmine Bromide	Tablet	17	7	6	YES
14	Nitroglycerin	Tablet	17	7	13	YES
15	Ethacridine	Injection	17	2	5	YES
16	Noradrenaline/Norepinephrine	Injection	16	2	7	YES
17	Isoprenaline	Injection	16	1	3	YES
18	Cytarabine	Injection	15	3	8	YES
19	Allopurinol	Tablet	15	2	21	YES
20	Calcium gluconate	Injection	15	3	14	YES
21	Propafenone	Injection	15	4	43	YES
22	Adrenaline	Injection	15	13	5	YES
23	Dobutamine	Injection	14	3	8	YES
24	Mitoxantrone	Injection	14	3	7	YES
25	Benzathine Benzylpenicillin	Injection	14	3	25	YES
26	Pralidoxime Chloride	Injection	14	4	4	YES
27	Phentolamine	Injection	13	5	9	YES
28	Mitomycin	Injection	13	1	4	YES
29	Ketamine	Injection	13	2	8	NO
30	Bleomycin A5	Injection	13	1	3	YES
31	Phenobarbital	Injection	12	3	6	YES
32	Sodium Thiosulfate	Injection	12	0	4	YES
33	Oxytocin	Injection	12	4	21	YES
34	Verapamil	Injection	12	2	7	YES
35	Metaraminol	Injection	12	1	12	NO
36	Adrenocorticotropine	Injection	11	1	2	YES
37	Diazepam	Injection	11	4	20	YES
38	Dopamine	Injection	11	2	9	YES
39	Furosemide	Injection	11	3	58	YES
40	Magnesium Sulfate	Injection	11	5	19	YES
41	Vincristine	Injection	11	3	10	YES
42	Thiamazole	Tablet	10	1	16	YES
43	Arginine	Injection	10	12	13	YES
44	Acetamide	Injection	10	0	1	YES
45	Oryzanol	Tablet	10	3	200	NO
46	Tetanus antitoxin	Injection	10	0	2	NO
47	Hydroxycarbamide	Tablet	10	1	5	NO
48	Cyclophosphamide	Injection	9	5	4	YES
49	Methylene Blue	Injection	9	1	3	YES
50	Pralidoxime Iodide	Injection	9	2	7	NO
51	Chlorpromazine	Injection	9	4	28	NO
52	Phenylephrine	Injection	9	1	2	NO
53	Nystatin	Tablet	9	2	15	NO
54	Sodium Dimercaptopropane Sulfonate	Injection	8	1	3	YES
55	Hydrocortisone	Injection	8	7	43	YES
56	Mercaptopurine	Tablet	8	3	5	YES
57	Iodinated Oil	Injection	8	2	3	NO
58	Scopolamine	Injection	8	2	9	NO
59	Promethazine	Injection	8	4	24	NO
60	Penicillamine	Tablet	7	2	4	YES
61	Amiodarone	Injection	7	2	4	YES
62	Digoxin	Oral liquid	7	0	1	YES
63	Etoposide	Injection	7	3	14	YES
64	Haloperidol	Injection	7	2	6	NO
65	Heparin	Injection	7	12	18	NO
66	Suxamethonium Chloride	Injection	7	2	3	NO
67	Chymotrypsin	Injection	7	1	4	NO
68	Chorionic Gonadotrophin	Injection	7	4	14	NO
69	Sodium Bicarbonate	Tablet	7	4	124	NO
70	Clofazimine	Capsule	6	1	1	YES
71	Thrombin	Lyophilizing Powder	6	2	20	YES
72	Sodium Nitroprusside	Injection	6	5	12	YES
73	Aminophylline	Injection	6	7	45	NO
74	Bupivacaine	Injection	6	2	9	NO
75	Estriol	Cream	6	0	1	NO
76	Etamsylate	Injection	6	5	57	NO
77	Metoclopramide	Injection	6	2	15	NO
78	Labetalol	Injection	6	2	2	NO
79	Propranolol	Tablet	6	4	58	NO
80	Human albumin	Injection	6	0	44	NO
81	Isoniazid	Injection	6	4	18	NO
82	Testosterone Undecanoate	Capsule	6	1	2	NO
83	Stanozolol	Tablet	6	2	4	NO
84	Aminophylline	Tablet	5	7	135	NO
85	Testosterone Propionate	Injection	5	3	4	NO
86	Progesterone	Injection	5	11	12	NO
87	Metoclopramide	Tablet	5	2	71	NO
88	Carbidopa and Levodopa	Controlled/Sustained-release Tablet	5	0	1	NO
89	Rifapentine	Capsule	5	2	6	NO
90	Procaine	Injection	5	5	95	NO
91	Anisodamine	Tablet	5	1	2	NO
92	Folic acid	Tablet	5	3	29	NO
93	Dextran 40 Sodium Chloride	Injection	5	0	26	NO
94	Aminomethylbenzoic Acid	Injection	5	5	36	NO
95	Menadione Sodium Bisulfite	Injection	5	5	23	NO

^*^Number of occurrences in different years in different provinces

### Regional characteristics of drug shortages

Except for Anhui Province, Fujian Province, Henan Province, Zhejiang Province, Qinghai Province, Tibet Autonomous Region and Chongqing Municipality, a total of 24 provinces issued a list of drug shortages in the past four years (Table 2). In terms of the average number of annual drugs in shortage, the western region is the most serious (41.67 ± 29.55), while the northeast region is the least serious (18.67 ± 4.04). However, there is no significant difference in drug shortages among economic regions (F = 0.829, P = 0.493 > 0.05).

Gansu Province leads the list with a shortage of 106 drugs/year on average. Sichuan Province has the fewest shortage, with only 6 drugs.

**Table 2:** Drug shortages in provinces in China, 2018–2021

NO.	Provinces	economic area	Number of drugs in short supply				Total*	Drugs/year
			2018	2019	2020	2021		
1	Gansu province	Western	150	61	UR	UR	211	106
2	Guangxi Zhuang Autonomous Region	Central	39	65	UR	62	166	72
3	Shanghai	Eastern	37	38	29	37	141	58
4	Tianjin	Western	UR	20	UR	69	89	57
5	Yunnan province	Western	68	UR	UR	10	78	55
6	Hubei province	Eastern	UR	UR	UR	72	72	45
7	Jiangsu province	Western	UR	UR	25	44	69	42
8	Ningxia Hui Autonomous Region	Western	41	UR	25	UR	66	39
9	Hunan province	Eastern	41	UR	26	UR	67	36
10	Beijing	Eastern	UR	UR	58	UR	58	35
11	Inner Mongolia Autonomous Region	Eastern	57	UR	UR	UR	57	35
12	Guizhou province	Central	UR	24	14	13	51	34
13	Hainan province	Western	UR	UR	22	31	53	33
14	Xinjiang Uygur Autonomous Region	Eastern	UR	UR	UR	42	42	29
15	Liaoning province	Eastern	UR	UR	25	20	45	27
16	Shaanxi province	Eastern	UR	18	22	UR	40	26
17	Shanxi Province	North East	UR	UR	33	8	41	23
18	Hebei province	Central	36	UR	UR	UR	36	21
19	Guangdong province	Western	UR	26	UR	UR	26	20
20	Shandong province	Central	UR	UR	UR	29	29	20
21	Jilin province	North East	UR	UR	18	UR	18	18
22	Jiangxi province	Western	UR	UR	UR	20	20	17
23	Heilongjiang province	North East	UR	UR	15	UR	15	15
24	Sichuan province	Western	UR	UR	UR	6	6	6
	Total		469	252	312	463	1496	374

^*^Duplicate drugs were not excluded; UR: Unreleased

### Therapeutic categories of drug shortages

Among 408 drugs, chemical medicine accounted for 94.1%, while traditional Chinese medicine accounted for 5.9%. According to the principles of the ATC classification of the WHO, chemical medicine shortages included 14 categories, with cardiovascular drugs, nervous system drugs, antineoplastic and immunomodulating agents, and drugs for blood and blood forming organs over 10% (Table 3). The most common cardiovascular drugs in shortage were nitroglycerin, epinephrine, and norepinephrine.

**Table 3:** The therapeutic categories of chemical medicine in shortage in China, 2018–2021

No.	Therapeutic category	Number of drugs	Frequency in the provincial shortage lists	Proportion^*^ (%)
1	Cardiovascular system	42	254	17.0%
2	Nervous system	53	204	13.6%
3	Antineoplastic and immunomodulating agents	31	181	12.1%
4	Blood and blood forming organs	42	159	10.6%
5	Alimentary tract and metabolism	42	128	8.6%
6	Anti-infectives for systemic use	47	111	7.4%
7	Various	21	105	7.0%
8	Genito urinary system and sex hormones	24	84	5.6%
9	Systematic hormonal preparations, sex hormones and insulins	14	77	5.1%
10	Respiratory system	18	76	5.1%
11	Dermatologicals	18	39	2.6%
12	Musculo-skeletal system	7	36	2.4%
13	Sensory organs	18	30	2.0%
14	Antiparasitic products, insecticides and repellents	7	12	0.8%

^*^Proportion of frequency of drug shortages

### Drug shortages in China on each list

Among 408 drugs that have experienced shortages, the essential medicines in NEML and WHO Model List accounted for 51.0% (208/408) and 67.9% (277/408), respectively, with 25.5% (104/408) in both lists, including neostigmine, pyridostigmine bromide, ketamine, phenobarbital sodium, diazepam, magnesium sulfate, etc. The complete information on 104 drugs is in Table S2 of Supplementary Material.

Compared with NMIDL and VBPL, 81.9% (334/408) of drugs in shortage fell in NMIDL, with 54.9% (224/408) in category A and 27.0% (110/408) in category B. Only 3.4% (14/408) was in VBPL (Table S3 in Supplementary Material).

The analysis of variance showed that the drug shortage incidence of NEML, the WHO Model List of Essential Medicines and Medical insurance category A was significantly higher than that of medical insurance category B and VBPL (P < 0.05), (Table S3 in Supplementary Material)

Notably, 72.0% (36/50) of the drugs in EDL experienced shortages (Table 4). It was significantly higher than all other lists (P < 0.05).

**Table 4:** Emergency drugs in shortage in China, 2018–2021

No.	Drug name	Dosage form
1	Dopamine	Injection
2	Norepinephrine	Injection
3	Metaraminol	Injection
4	Phentolamine	Injection
5	Sodium Nitroprusside	Injection
6	Nitroglycerin	Injection
7	Isosorbide nitrate	Injection
8	Esmolol	Injection
9	Metoprolol	Injection
10	Propafenone	Injection
11	Heparin Sodium	Injection
12	Heparin Calcium	Injection
13	Urokinase	Injection
14	Protamine	Injection
15	Tranexamic Acid	Injection
16	Amino caproic Acid	Injection
17	Posterior Pituitary	Injection
18	Mannitol	Injection
19	Succinylcholine Chloride	Injection
20	Haloperidol	Injection
21	Magnesium Sulfate	Injection
22	Neostigmine	Injection
23	Pralidoxime Chloride	Injection
24	Pralidoxime Iodide	Injection
25	Sodium Thiosulfate	Injection
26	Naloxone	Injection
27	Methylene Blue	Injection
28	Acetamide	Injection
29	Sodium Dimercaptopropyl Sulfonate	Injection
30	Antivenin	Injection
31	Tetanus Antitoxin	Injection
32	Tetanus Human Immunoglobulin	Injection
33	Glucose	Injection
34	Sodium Bicarbonate	Injection
35	Dextran (40) Sodium Chloride	Injection
36	Dextran (40) Glucose	Injection

## Discussions

In this study, 408 drugs have been in shortage in the last four years in China. Meanwhile, like the United States, France, and other countries [5, 8, 26], China’s drug shortage has shown an upward trend in recent years. It indicated the drug shortage’s severity.

Of the 671 medicines in shortage in Europe, including Belgium, the Netherlands, the United Kingdom, Italy, Germany, Spain, and France from 2010 to 2013, 51% were oral, and 40% were injection, but 52% were injectable, and 40% were oral among 200 essential medicines [27]. In France, injections and oral agents accounted for 47.5% and 43.3% of the 3,530 drugs in shortage during the 7-year period from 2012 to 2018 [5]. In the United States, most drugs affected by shortages from 2014 to 2019 were parenteral (61.2%), followed by oral (25.4%) [4]. Consistent with our findings, the largest proportion of drugs in shortage were injections. The underlying cause of many drug shortages is a lack of profit due to low prices, in which case injections are likelier to be in shortage due to their higher cost than other dosage forms [28].

Drug shortages causes are multifactorial. The drug shortages caused by raw material shortages and production issues have been considered globally [29]. Moreover, the number of hospital patients, as a variable significantly affecting shortage frequency, may indicate that the great demand for drugs will also lead to the emergence of shortages [11]. Low margins, rather than low prices, lack of incentives for high-quality drugs in the generic market, and complexity of drug logistics and regulations further complicate restoring the supply after the market disruptions [28, 30]. As indicated by this study, 3 to 50 manufacturers produced 76.8% (73/95) of the drugs in shortage with a frequency ≥ 5. The lack of manufacturers could not explain the shortage of all drugs. However, some drugs were in shortage and APIs were only produced by fewer than three manufacturers, indicating a potential risk of supply interruption and needed to be closely monitored. To solve this problem, fixed-point productions for some shortages of drugs were conducted in China. The Chinese government invites bids from manufacturers for drugs that are clinically necessary and shortages, and the bid-winning enterprises will conduct fixed-point production. The government-run primary medical and health institutions and public hospitals are required to be equipped with fixed-point production varieties and pay in time according to the contract. At the same time, other medical and health institutions are encouraged to purchase and use designated production varieties. Relevant departments shall monitor the production and supply of designated production enterprises, coordinate and solve existing problems, and ensure stable production and effective supply [31]. Furthermore, regulatory issues such as lack of monitoring systems or policies and unavailability of communication among stakeholders can also affect drug shortages [2]. National and provincial monitoring systems for drug shortages have been established in China since 2016 [32]. However, drug shortage information was mostly reported by medical institutions, and reporting of drug manufacturers was generally insufficient, resulting in the delay of warning for 3 to 6 months [33, 34]. A multi-source information collection platform for drug shortages was launched in November 2021 to realize information interconnection and sharing among drug manufacturers, distributors, and medical institutions; facilitate the joint response to shortages; and improve the sensitivity and timeliness of monitoring and response. It is a national-level platform for monitoring and early warning. Based on this platform, we suggest establishing a shared drug inventory database to place the warning even earlier. Demand issues also causes of drug shortages. Emergency drugs, such as protamine, pralidoxime chloride, and sodium thiosulfate are likely to be in short supply due to low market demand. Yang et al. proposed establishing a reserve system for drug shortages to reserve specific medicines, such as emergency medicines and orphan drugs [10].

Unlike our study, Yang et al. showed that there was no significant difference in the frequency of drug shortages in different regions, but that the number of emergency medicine shortages in eastern regions was significantly higher than the number of shortages in western and central regions [11]. The difference may be because it only investigated the shortages of emergency medicine and the number of drugs in shortage or because the scope of the investigation was significantly narrower than in our study. In general, according to our study, some drugs were in shortage nationwide and others only in several specific provinces. It presented the characteristics of national shortages accompanied by regional shortages in China. This may be related to the differences in disease distribution [35]. Local medicine policies, drug inventory, economic development, and transportation convenience, as well as the pharmaceutical industry, may also influence drug supply. Studies have shown that the drug supply in central China is better than in eastern and western China [36].

In terms of therapeutic properties, nearly all categories of drugs had been reported to be in short supply. The distribution of therapeutic categories for drug shortages in China was similar to other countries such as the United States, Europe and Australia, and was an international public health challenge. In many countries, including China, the cardiovascular system, nervous system, antineoplastic and immunomodulating agents and Anti-infectives for systemic use are among the top categories with the most serious shortage [5, 27, 37]. As the first-line therapy for several cardiovascular diseases, the shortage of antianginals, inotropes, and vasopressors may result in serious health consequences, such as an unsatisfactory effect or increased risk of side effects [38]. An upward trend was also observed in the number of cardiovascular drugs in short supply reported each year in the United States, according to the UUDIS database, from 2017 to 2021 [6]. Ongoing shortages of cardiovascular drugs may have a major impact on public health. Neostigmine, the most commonly used acetylcholinesterase inhibitor in perioperative medicine, was the most common nervous system drug in shortage not only in China but also in the United States [39]. Similarly, pyridostigmine bromide was also in short supply. The shortage of antineoplastic drugs was a particular challenge. There are also shortages of many drugs that played an important role in cancer treatment, such as methotrexate, bleomycin, cytarabine and mitoxantrone. The previous studies reported increased tumor recurrence and impaired survival in cancer patients treated with non-standard therapy during shortages [40]. In addition, there was also a high risk of shortages of blood system drugs such as vitamin K1 and urokinase in China according to this study as well as the previous study [41].

The results of a previous survey of 236 emergency department physicians showed that most respondents agreed that original medicines, injections, essential medicines, medicines without alternative agents, and cheap medicines were more susceptible to shortages than generics, orals, nonessential medicines, medicines with alternative agents and expensive medicines, respectively [11]. In China, 30.4% of essential medicines are in shortage, accounting for 51.0% of the shortage. China had severe essential medicine shortages, consistent with previous surveys in other countries. In a European survey, 30% of drugs with reported shortages were the WHO essential medicines [27]. Shortages of essential medicines have also been reported frequently in some countries in North America, Asia, and South America [42–44]. Global access to essential medicines remains problematic.

Furthermore, most drugs in shortage were medical insurance drugs in our study. Essential medicines and medical insurance drugs have the characteristics of clinical necessity, safety, effectiveness, reasonable price and guaranteed quality. Therefore, more attention should be paid to these drugs’ shortages. The supply guarantee of such drugs is highly significant to ensure basic medical services for the public.

Consistent with other studies [45], we also found that emergency drugs were likelier to be in short supply. If these drugs are unavailable, the common clinical effect of emergency drug shortages on patients is to delay treatment [11]. Patients may miss the best treatment opportunity and face serious risks if these drugs are unavailable.

This study also found that drug shortage incidence (5.9%) and the VBPL proportion in the drug shortage list (3.4%) were very low, significantly lower than other lists. Therefore, the national procurement pattern of VBPL may effectively decrease drug shortages. This pattern realized the procurement alliance and the inter-provincial adjustment. Its biggest advantage is to ensure the number of transactions and the government will pay a certain percentage of the transaction as the advance payment to guarantee enterprises’ production, so the unit price is low and the total profit of enterprises is sufficient. In the United States, the European Union and some other countries, extensive research has been conducted on mitigation strategies. Many measures have been proposed to cope with drug shortages. In addition to the above measures, risk assessment procedures, national mitigation guidelines, public service obligations on drug suppliers, expediting of drug reviews to restore production, and other measures have good reference significance for the management of drug shortages in China [46–48].Generally, drug shortages are a global problem that requires international collaboration to develop global mitigation strategies. In high-income countries, almost all categories of drugs have experienced shortages during different periods. In low- and middle-income countries, previous studies mainly focused on the affordability/availability of some essential medicines [2]. Compared to the United States and European countries, less research on drug shortages has been conducted in China. The goal of this study is to comprehensively collect and analyze the national and provincial lists of drug shortages issued in China over the past four years. This will provide references for solving the problem.

Some limitations to this study should be noted. Due to the lack of data in many provinces, there may be some bias in this study. The ATC classifications and manufacturers of the drugs in the list were manually matched. Due to the short implementation time and the absence data from seven provinces, our study was not able to adequately analyze drug shortage trends and assess the effects of new policies or measures. Further research is needed on the causes, trends and effects of intervention.

## Conclusion

The overview of drug shortages in China was summarized and analyzed in this study. Drug shortages in China has been severe and complicated, and the shortage of injections has been the worst. The number of drugs in shortage was different but not significant among different economic regions, among which the western region had the most shortages, and the northeast region had the fewest shortages. The incidence of drug shortages on the Volume-Based Purchasing List is significantly lower than that of the WHO Model List of Essential Medicines, NEML, NMIDL and EDL. National procurement pattern of volume-based drug purchasing may be conducive to alleviating the problem of drug shortages. The collaboration of all partners was recommended to ensure the supply of clinically necessary drugs. This study enabled all practitioners to comprehensively understand the shortage of drugs in China and provided references for improvement.

## Electronic supplementary material

Below is the link to the electronic supplementary material.

Supplementary Material 1

## Data Availability

All data generated or analysed during this study are included in this published article [and its supplementary information files]. Available from: https://pan.baidu.com/s/172gZVYpihErTZ3oryk5HxA, the extracted code is 3d2y.

## Abbreviations

NMPA: National Medical Products Administration
WHO: World Health Organization
NEML: National Essential Medicine List
NMIDL: National Medical Insurance Drug List
API: Active pharmaceutical ingredient
ATC: Anatomical Therapeutic Chemical
EDL: Emergency Drug List
VBPL: Volume-Based Purchasing List

## References

[1] Gray A, Manasse HR (2012). Shortages of medicines: a complex global challenge. Bull World Health Organ.

[2] Shukar S, Zahoor F, Hayat K, Saeed A, Gillani AH, Omer S, Hu S, Babar ZU, Fang Y, Yang C (2021). Drug shortage: causes, impact, and mitigation strategies. Front Pharmacol.

[3] Report M. Technical Definitions of Shortages and Stockouts of Medicines and Vaccines [https://www.who.int/publications/m/item/WHO-EMP-IAU-2017.03]

[4] Patel R, Samiee-Zafarghandy S, Ziesenitz V, Fox ER, Van Den Anker J, Ong H, Mazer-Amirshahi M (2022). US drug shortages compared to the World Health Organization’s Model list of essential Medicines for Children: a cross-sectional study. Am J Health Syst Pharm.

[5] Benhabib A, Ioughlissen S, Ratignier-Carbonneil C, Maison P (2020). The french reporting system for drug shortages: description and trends from 2012 to 2018: an observational retrospective study. BMJ Open.

[6] Drug Shortages Statistics [https://www.ashp.org/Drug-Shortages/Shortage-Resources/Drug-Shortages-Statistics?loginreturnUrl=SSOCheckOnly]

[7] Biedermann F (2022). New Dutch regulations to alleviate drug shortages. Lancet.

[8] De La Sabogal ML, Tucker EL (2022). Drug shortages in low- and middle-income countries: Colombia as a case study. J Pharm Policy Pract.

[9] The Administration Rules (trial) of the National List of Drug Shortages. [http://www.gov.cn/zhengce/zhengceku/2020-04/24/5505943/files/cfab9e4b70fe414495bca16dbae50df1.pdf]. (in Chinese)]

[10] Yang C, Wu L, Cai W, Zhu W, Shen Q, Li Z, Fang Y (2016). Current Situation, determinants, and solutions to drug shortages in Shaanxi Province, China: a qualitative study. PLoS ONE.

[11] Yang C, Cai W, Li Z, Page AT, Fang Y (2018). The current status and effects of emergency drug shortages in China: perceptions of emergency department physicians. PLoS ONE.

[12] Fan JWZ, ZY, Han S, Shi L, Gun S (2018). Analysis of drug shortage in China’s hospitals. Chin J New Drug.

[13] Notice on issuing the National List of Drug Shortages. [http://www.nhc.gov.cn/yaozs/s7653/202012/f30aad8ec4ba48a9afa2e559f4d20e7c.shtml]. (in Chinese)]

[14] Mazer-Amirshahi M, Pourmand A, Singer S, Pines JM, van den Anker J (2014). Critical drug shortages: implications for emergency medicine. Acad Emerg Med.

[15] Fox ER, Sweet BV, Jensen V (2014). Drug shortages: a complex health care crisis. Mayo Clin Proc.

[16] National Essential Medicine List. [http://www.nhc.gov.cn/ewebeditor/uploadfile/2018/10/20181025183346942.pdf]. (in Chinese)]

[17] WHO model list of essential medicines – 22nd list., 2021 [https://www.who.int/publications/i/item/WHO-MHP-HPS-EML-2021.02]

[18] Yip W, Fu H, Chen AT, Zhai T, Jian W, Xu R, Pan J, Hu M, Zhou Z, Chen Q (2019). 10 years of health-care reform in China: progress and gaps in Universal Health Coverage. Lancet.

[19] Zuo W, Mei D, Sun W, Tang X, Niu Z, Gao D, Zhang B (2020). The interpretation of China national essential medicines list 2018. Expert Rev Clin Pharmacol.

[20] Interim Measures for Medication Administration of Basic Medical Insurance. [http://www.nhsa.gov.cn/art/2020/7/31/art_37_3387.html]. (in Chinese)]

[21] Notice on printing and distributing the Medicine List for National Basic Medical Insurance., Industrial Injury Insurance and Maternity Insurance [http://www.nhsa.gov.cn/art/2021/12/3/art_37_7429.html]. (in Chinese)]

[22] Chen Y, Ji X, Xiao H, Unger JM, Cai Y, Mao Z, Yeung K (2021). Impact of the pilot volume-based Drug Purchasing Policy in China: interrupted time-series analysis with controls. Front Pharmacol.

[23] WHO model list. of essential medicines – 22nd list [https://www.who.int/publications/i/item/WHO-MHP-HPS-EML-2021.02]

[24] Model drugs for emergency (rescue) drugs purchased directly online [http://www.nhc.gov.cn/yaozs/s7652/201509/105a04136fad412e9f57fa8034126bd9.shtml]

[25] Communiqué on the Fourth National Economic Census. (No. 7) [http://www.stats.gov.cn/english/PressRelease/201911/t20191120_1710332.html]

[26] Patel R, Samiee-Zafarghandy S, Ziesenitz V, Fox ER, Van Den Anker J, Ong H, Mazer-Amirshahi M (2022). US drug shortages compared to the World Health Organization’s model list of essential Medicines for Children: a cross-sectional study. Am J Health-System Pharm.

[27] Pauwels K, Huys I, Casteels M, Simoens S (2014). Drug shortages in european countries: a trade-off between market attractiveness and cost containment?. BMC Health Serv Res.

[28] Hernandez I, Hershey TB, Donohue JM (2020). Drug shortages in the United States: are some prices too low?. JAMA.

[29] Bogaert P, Bochenek T, Prokop A, Pilc A (2015). A qualitative Approach to a better understanding of the problems underlying drug shortages, as viewed from belgian, french and the European Union’s perspectives. PLoS ONE.

[30] Shuman A, Unguru Y (2020). Drug shortages: the View Across an Ocean. Oncologist.

[31] Notice on matters related to the. fixed-point production pilot of clinically necessary drugs, low useage and shortage of market supply in 2016 [http://www.nhc.gov.cn/yaozs/s3581/201701/75150182dbb74ca78ce3397d28d51cb8.shtml] (in Chinese)]

[32] Notice of the General Office of the National Health and Family Planning Commission on. the establishment of a pilot project for monitoring and reporting of shortages of drugs [http://www.jxwst.gov.cn/doc/2016/05/11/64731.shtml. (in Chinese)]

[33] Ma LMY, Wang H, Zhao Y, Li S, Huang P (2020). Study on the Causes and coping strategies of drug shortage in Hubei Province. Med Soc.

[34] Mao NZM, Li J (2018). Analysis on the cause of drug shortage in China from the perspective of economics. China J Hosp Pharm.

[35] Zhou M, Wang H, Zeng X, Yin P, Zhu J, Chen W, Li X, Wang L, Wang L, Liu Y (2019). Mortality, morbidity, and risk factors in China and its provinces, 1990–2017: a systematic analysis for the global burden of Disease Study 2017. Lancet.

[36] Xie X, Ellis A, Wang Y, Xie Z, Duan M, Su C (2009). Geochemistry of redox-sensitive elements and sulfur isotopes in the high arsenic groundwater system of Datong Basin, China. Sci Total Environ.

[37] Cameron EE, Bushell MA (2021). Analysis of drug shortages across two countries during pre-pandemic and pandemic times. Res Social Adm Pharm.

[38] Reed BN, Fox ER, Konig M, Jackevicius CA, Masoudi FA, Rabinstein AA, Page RL (2016). The impact of drug shortages on patients with cardiovascular disease: causes, consequences, and a call to action. Am Heart J.

[39] Shaydenfish D, Wongtangman K, Eikermann M, Schaefer MS (2020). The effects of acetylcholinesterase inhibitors on morbidity after general anesthesia and surgery. Neuropharmacology.

[40] Goldsack JC, Reilly C, Bush C, McElligott S, Bristol MN, Motanya UN, Field R, Vozniak JM, Wong YN, Schwartz JS (2014). Impact of shortages of injectable oncology drugs on patient care. Am J Health Syst Pharm.

[41] Shi Y, Sun S, Deng J, Liu S, Yin T, Peng Q, Gong Z, Cheng Z, Zhou B. Establishment and Application of an Index System for the Risk of Drug Shortages in China: Based on Delphi Method and Analytic Hierarchy Process.Int J Health Policy Manag2022. 10.34172/ijhpm.2022.6360 PMC10105199 35297233

[42] Atif M, Malik I, Mushtaq I, Asghar S (2019). Medicines shortages in Pakistan: a qualitative study to explore current situation, reasons and possible solutions to overcome the barriers. BMJ Open.

[43] Chebolu-Subramanian V, Sundarraj RP (2021). Essential medicine shortages, procurement process and supplier response: a normative study across Indian states. Soc Sci Med.

[44] Situation of Essential Medicines at Risk of Supply Shortage with Emphasis on South American Countries. [http://isags-unasur.org/en/publicacao/situation-of-essential-medicines-at-risk-of-supply-shortage-with-emphasis-on-south-american-countries-2/]

[45] Lin MP, Vargas-Torres C, Shin-Kim J, Tin J, Fox E (2022). Nearly all thirty most frequently used emergency department drugs experienced shortages from 2006–2019. Am J Emerg Med.

[46] Miljković N, Godman B, Kovačević M, Polidori P, Tzimis L, Hoppe-Tichy T, Saar M, Antofie I, Horvath L, De Rijdt T (2020). Prospective risk Assessment of Medicine shortages in Europe and Israel: findings and implications. Front Pharmacol.

[47] Fox ER, McLaughlin MM (2018). ASHP guidelines on managing drug product shortages. Am J Health Syst Pharm.

[48] Bochenek T, Abilova V, Alkan A, Asanin B, de Miguel Beriain I, Besovic Z, Vella Bonanno P, Bucsics A, Davidescu M, De Weerdt E (2017). Systemic measures and legislative and organizational frameworks aimed at preventing or mitigating drug shortages in 28 european and western asian countries. Front Pharmacol.

