# Supplementary material for: Drug shortages in China: a cross-sectional study
Source: BMC Health Serv Res. 2023 May 4;23:438. doi: 10.1186/s12913-023-09295-w (PMC10159680; doi:10.1186/s12913-023-09295-w)
Supplement: Supplementary file 1 — Supplementary Material 1 [file 12913_2023_9295_MOESM1_ESM.docx]

Additional file 1

**Table S1.** Drugs in the provincial lists of drug shortages in China, 2018-2021.

| Ranking | Name | Dosage form | Provincial lists of drug shortages | Therapeutic category | Frequency in the provincial shortage lists^*^ | Whether in the national list of drug shortages |
| --- | --- | --- | --- | --- | --- | --- |
| 1 | Methotrexate | Injection | Beijing 2020, Hainan province 2021, Hubei province 2021, Guangxi Zhuang Autonomous Region 2021, Shanxi Province 2021, Shanghai 2021, Tianjin 2021, Xinjiang Uygur Autonomous Region 2021, Yunnan province 2021, Guizhou province 2020, Hainan province 2020, Heilongjiang province 2020, Shanxi Province 2020, Shaanxi province 2020, Hunan province 2020, Ningxia Hui Autonomous Region 2020, Gansu province 2019, Guangdong province 2019, Guangxi Zhuang Autonomous Region 2019, Guizhou province 2019, Shaanxi province 2019, Shanghai 2019, Guangxi Zhuang Autonomous Region 2018, Hunan province 2018, Inner Mongolia Autonomous Region 2018, Ningxia Hui Autonomous Region 2018 | Antineoplastic and immunomodulating agents | 26 | YES |
| 2 | Vitamin K | Injection | Beijing 2020, Guangxi Zhuang Autonomous Region 2021, Hainan province 2021, Hubei province 2021, Tianjin 2021, Shanghai 2021, Xinjiang Uygur Autonomous Region 2021, Hainan province 2020, Guizhou province 2020, Jilin province 2020, Hunan province 2020, Liaoning province 2020, Ningxia Hui Autonomous Region 2020, Shaanxi province 2020, Shanxi Province 2020, Shanghai 2020, Guangxi Zhuang Autonomous Region 2018, Hunan province 2018, Inner Mongolia Autonomous Region 2018, Ningxia Hui Autonomous Region 2018, Shanghai 2018, Yunnan province 2018 | Blood and blood forming organs | 22 | YES |
| 3 | Posterior pituitary | Injection | Beijing 2020, Guangxi Zhuang Autonomous Region 2021, Hainan province 2021, Tianjin 2021, Hubei province 2021, Jiangsu province 2021, Shandong province 2021, Shanghai 2021, Xinjiang Uygur Autonomous Region 2021, Yunnan province 2021, Guizhou province 2020, Hainan province 2020, Jiangsu province 2020, Liaoning province 2020, Shanxi Province 2020, Guangxi Zhuang Autonomous Region 2019, Guizhou province 2019, Shanghai 2019, Hunan province 2018, Inner Mongolia Autonomous Region 2018, Ningxia Hui Autonomous Region 2018 | Systematic hormonal preparations, sex hormones and insulins | 21 | YES |
| 4 | Urokinase | Injection | Beijing 2020, Guizhou province 2021, Guangxi Zhuang Autonomous Region 2021, Hainan province 2021, Hubei province 2021, Hunan province 2020, Jiangsu province 2021, Jiangxi province 2021, Liaoning province 2021, Tianjin 2021, Shandong province 2021, Shanghai 2021, Yunnan province 2021, Hainan province 2020, Hunan province 2020, Ningxia Hui Autonomous Region 2020, Shaanxi province 2020, Shanghai 2020, Guangdong province 2019, Shaanxi province 2019, Shanghai 2018 | Blood and blood forming organs | 21 | YES |
| 5 | Protamine | Injection | Beijing 2020, Guangxi Zhuang Autonomous Region 2021, Guizhou province 2021, Hainan province 2021, Hubei province 2021, Jiangxi province 2021, Shandong province 2021, Tianjin 2021, Xinjiang Uygur Autonomous Region 2021, Guangxi Zhuang Autonomous Region 2021, Hainan province 2020, Jiangsu province 2020, Shaanxi province 2020, Hunan province 2020, Ningxia Hui Autonomous Region 2020, Guangxi Zhuang Autonomous Region 2019, Guangdong province 2019, Guangxi Zhuang Autonomous Region 2018, Shaanxi province 2019, Inner Mongolia Autonomous Region 2018, Yunnan province 2018 | Various | 21 | YES |
| 6 | Atropine | Injection | Beijing 2020, Hubei province 2021, Jiangsu province 2021, Tianjin 2021, Xinjiang Uygur Autonomous Region 2021, Hunan province 2020, Jiangsu province 2020, Ningxia Hui Autonomous Region 2020, Shanghai 2020, Gansu province 2019, Guangdong province 2019, Shanghai 2019, Tianjin 2019, Gansu province 2018, Guangxi Zhuang Autonomous Region 2018, Hebei province 2018, Inner Mongolia Autonomous Region 2018, Ningxia Hui Autonomous Region 2018, Shanghai 2018, Yunnan province 2018 | Alimentary tract and metabolism | 20 | YES |
| 7 | Lobeline | Injection | Beijing 2020, Guangxi Zhuang Autonomous Region 2021, Guizhou province 2021, Hubei province 2021, Jiangsu province 2021, Jiangxi province 2021, Liaoning province 2021, Tianjin 2021, Xinjiang Uygur Autonomous Region 2021, Guangxi Zhuang Autonomous Region 2021, Jilin province 2020, Jiangsu province 2020, Guangxi Zhuang Autonomous Region 2019, Gansu province 2019, Hebei province 2018, Inner Mongolia Autonomous Region 2018, Ningxia Hui Autonomous Region 2018, Shanghai 2018, Yunnan province 2018 | Respiratory system | 19 | YES |
| 8 | Bleomycin | Injection | Beijing 2020, Guangxi Zhuang Autonomous Region 2021, Guizhou province 2021, Hainan province 2021, Hubei province 2021, Jiangxi province 2021, Liaoning province 2021, Shandong province 2021, Shanxi Province 2021, Tianjin 2021, Hainan province 2020, Hunan province 2020, Jiangsu province 2021, Ningxia Hui Autonomous Region 2020, Shanxi Province 2020, Shaanxi province 2020, Gansu province 2019, Guangxi Zhuang Autonomous Region 2019, | Antineoplastic and immunomodulating agents | 18 | YES |
| 9 | Deslanoside | Injection | Beijing 2020, Hubei province 2021, Jiangsu province 2021, Jiangxi province 2021, Tianjin 2021, Xinjiang Uygur Autonomous Region 2021, Jilin province 2020, Jiangsu province 2020, Hunan province 2020, Shanxi Province 2020, Gansu province 2019, Guangxi Zhuang Autonomous Region 2019, Tianjin 2019, Guangxi Zhuang Autonomous Region 2018, Hebei province 2018, Inner Mongolia Autonomous Region 2018, Ningxia Hui Autonomous Region 2018, Yunnan province 2018 | Cardiovascular system | 18 | YES |
| 10 | Nikethamide | Injection | Beijing 2020, Hainan province 2021, Hubei province 2021, Jiangsu province 2021, Tianjin 2021, Shanghai 2021, Xinjiang Uygur Autonomous Region 2021, Guangxi Zhuang Autonomous Region 2021, Hainan province 2020, Gansu province 2019, Guangdong province 2019, Tianjin 2019, Gansu province 2018, Inner Mongolia Autonomous Region 2018, Ningxia Hui Autonomous Region 2018, Shanghai 2018, Yunnan province 2018 | Respiratory system | 17 | YES |
| 11 | Nitroglycerin | Injection | Beijing 2020, Guangxi Zhuang Autonomous Region 2021, Hubei province 2021, Tianjin 2021, Jiangsu province 2021, Shanghai 2021, Xinjiang Uygur Autonomous Region 2021, Shanxi Province 2020, Guangxi Zhuang Autonomous Region 2019, Gansu province 2019, Tianjin 2019, Shanghai 2019, Gansu province 2018, Guangxi Zhuang Autonomous Region 2018, Inner Mongolia Autonomous Region 2018, Shanghai 2018, Yunnan province 2018 | Cardiovascular system | 17 | YES |
| 12 | Neostigmine | Injection | Beijing 2020, Hubei province 2021, Hunan province 2020, Liaoning province 2021, Shandong province 2021, Tianjin 2021, Xinjiang Uygur Autonomous Region 2021, Yunnan province 2021, Ningxia Hui Autonomous Region 2020, Shanghai 2020, Hunan province 2020, Liaoning province 2020, Shanxi Province 2020, Shaanxi province 2020, Gansu province 2019, Gansu province 2018, Inner Mongolia Autonomous Region 2018 | Nervous system | 17 | YES |
| 13 | Pyridostigmine Bromide | Tablet | Beijing 2020, Guangxi Zhuang Autonomous Region 2021, Hainan province 2021, Hubei province 2021, Shandong province 2021, Tianjin 2021, Liaoning province 2020, Ningxia Hui Autonomous Region 2020, Shanxi Province 2020, Guangdong province 2019, Guangxi Zhuang Autonomous Region 2019, Guizhou province 2019, Guangxi Zhuang Autonomous Region 2018, Hunan province 2018, Ningxia Hui Autonomous Region 2018, Shanghai 2018, Yunnan province 2018 | Nervous system | 17 | YES |
| 14 | Nitroglycerin | Tablet | Beijing 2020, Guangxi Zhuang Autonomous Region 2021, Hubei province 2021, Tianjin 2021, Hainan province 2021, Shanghai 2021, Guizhou province 2020, Hainan province 2020, Hunan province 2020, Jilin province 2020, Gansu province 2019, Guangxi Zhuang Autonomous Region 2019, Guizhou province 2019, Shanghai 2019, Inner Mongolia Autonomous Region 2018, Yunnan province 2018 | Genito urinary system and sex hormones | 17 | YES |
| 15 | Ethacridine | Injection | Beijing 2020, Guangxi Zhuang Autonomous Region 2021, Hubei province 2021, Tianjin 2021, Shandong province 2021, Shanxi Province 2021, Xinjiang Uygur Autonomous Region 2021, Heilongjiang province 2020, Shanxi Province 2020, Shanghai 2020, Gansu province 2019, Guangxi Zhuang Autonomous Region 2019, Guangdong province 2019, Guizhou province 2019, Shanghai 2019, Ningxia Hui Autonomous Region 2018, Yunnan province 2018 | Cardiovascular system | 17 | YES |
| 16 | Noradrenaline/Norepinephrine | Injection | Beijing 2020, Hubei province 2021, Shandong province 2021, Shanghai 2021, Tianjin 2021, Yunnan province 2021, Jiangsu province 2020, Shanxi Province 2020, Gansu province 2019, Guangdong province 2019, Guangxi Zhuang Autonomous Region 2019, Guizhou province 2019, Guangxi Zhuang Autonomous Region 2018, Inner Mongolia Autonomous Region 2018, Ningxia Hui Autonomous Region 2018, Yunnan province 2018 | Cardiovascular system | 16 | YES |
| 17 | Isoprenaline | Injection | Beijing 2020, Guangxi Zhuang Autonomous Region 2021, Hubei province 2021, Shandong province 2021, Tianjin 2021, Xinjiang Uygur Autonomous Region 2021, Guangxi Zhuang Autonomous Region 2021, Hunan province 2020, Ningxia Hui Autonomous Region 2020, Jiangsu province 2020, Guangxi Zhuang Autonomous Region 2019, Gansu province 2019, Gansu province 2018, Inner Mongolia Autonomous Region 2018, Ningxia Hui Autonomous Region 2018, Yunnan province 2018 | Cardiovascular system | 16 | YES |
| 18 | Cytarabine | Injection | Beijing 2020, Guangxi Zhuang Autonomous Region 2021, Shandong province 2021, Tianjin 2021, Hubei province 2021, Yunnan province 2021, Guizhou province 2020, Ningxia Hui Autonomous Region 2020, Shaanxi province 2020, Gansu province 2019, Guizhou province 2019, Guangxi Zhuang Autonomous Region 2019, Shaanxi province 2019, Shanghai 2019, Tianjin 2019 | Antineoplastic and immunomodulating agents | 15 | YES |
| 19 | Allopurinol | Tablet | Beijing 2020, Hubei province 2021, Guangxi Zhuang Autonomous Region 2021, Tianjin 2021, Xinjiang Uygur Autonomous Region 2021, Guizhou province 2020, Hunan province 2020, Ningxia Hui Autonomous Region 2020, Gansu province 2019, Guangxi Zhuang Autonomous Region 2018, Guizhou province 2019, Hebei province 2018, Hunan province 2018, Ningxia Hui Autonomous Region 2018, Yunnan province 2018 | Musculo-skeletal system | 15 | YES |
| 20 | Calcium gluconate | Injection | Beijing 2020, Guangxi Zhuang Autonomous Region 2021, Hubei province 2021, Tianjin 2021, Xinjiang Uygur Autonomous Region 2021, Hunan province 2020, Shanghai 2020, Guangxi Zhuang Autonomous Region 2019, Shanghai 2019, Guangxi Zhuang Autonomous Region 2018, Hunan province 2018, Inner Mongolia Autonomous Region 2018, Ningxia Hui Autonomous Region 2018, Shanghai 2018, Yunnan province 2018 | Alimentary tract and metabolism | 15 | YES |
| 21 | Propafenone | Injection | Beijing 2020, Guangxi Zhuang Autonomous Region 2021, Guizhou province 2021, Hainan province 2021, Hubei province 2021, Jiangsu province 2021, Tianjin 2021, Yunnan province 2021, Jiangsu province 2020, Shanxi Province 2020, Shaanxi province 2020, Guangxi Zhuang Autonomous Region 2019, Shaanxi province 2019, Tianjin 2019, Yunnan province 2018 | Cardiovascular system | 15 | YES |
| 22 | Adrenaline | Injection | Beijing 2020, Guangxi Zhuang Autonomous Region 2021, Hubei province 2021, Shandong province 2021, Tianjin 2021, Xinjiang Uygur Autonomous Region 2021, Shanghai 2021, Shanxi Province 2020, Guangxi Zhuang Autonomous Region 2019, Gansu province 2019, Shanghai 2019, Gansu province 2018, Inner Mongolia Autonomous Region 2018, Ningxia Hui Autonomous Region 2018, Yunnan province 2018 | Cardiovascular system | 15 | YES |
| 23 | Dobutamine | Injection | Beijing 2020, Guizhou province 2021, Hainan province 2021, Hubei province 2021, Tianjin 2021, Xinjiang Uygur Autonomous Region 2021, Hainan province 2020, Shanghai 2020, Gansu province 2019, Guangdong province 2019, Shanghai 2019, Gansu province 2018, Inner Mongolia Autonomous Region 2018, Shanghai 2018 | Cardiovascular system | 14 | YES |
| 24 | Mitoxantrone | Injection | Beijing 2020, Hubei province 2021, Jiangxi province 2021, Liaoning province 2021, Shandong province 2021, Shanxi Province 2021, Shanghai 2021, Tianjin 2021, Xinjiang Uygur Autonomous Region 2021, Yunnan province 2021, Heilongjiang province 2020, Shanxi Province 2020, Shaanxi province 2020, Tianjin 2019 | Antineoplastic and immunomodulating agents | 14 | YES |
| 25 | Benzathine Benzylpenicillin | Injection | Beijing 2020, Hainan province 2021, Hubei province 2021, Tianjin 2021, Xinjiang Uygur Autonomous Region 2021, Shandong province 2021, Shanghai 2021, Yunnan province 2021, Guizhou province 2020, Heilongjiang province 2020, Jilin province 2020, Liaoning province 2020, Shanxi Province 2020, Guangxi Zhuang Autonomous Region 2019 | Anti-infectives for systemic use | 14 | YES |
| 26 | Pralidoxime Chloride | Injection | Beijing 2020, Guangxi Zhuang Autonomous Region 2021, Hainan province 2021, Hubei province 2021, Shandong province 2021, Tianjin 2021, Heilongjiang province 2020, Jiangsu province 2020, Shanxi Province 2020, Guangxi Zhuang Autonomous Region 2019, Tianjin 2019, Guangxi Zhuang Autonomous Region 2018, Shanghai 2018, Ningxia Hui Autonomous Region 2018 | Various | 14 | YES |
| 27 | Phentolamine | Injection | Beijing 2020, Guangxi Zhuang Autonomous Region 2021, Hubei province 2021, Jiangsu province 2021, Shandong province 2021, Shanghai 2021, Tianjin 2021, Xinjiang Uygur Autonomous Region 2021, Jiangsu province 2020, Shanxi Province 2020, Gansu province 2018, Guangxi Zhuang Autonomous Region 2019, Inner Mongolia Autonomous Region 2018 | Cardiovascular system | 13 | YES |
| 28 | Mitomycin | Injection | Beijing 2020, Guangxi Zhuang Autonomous Region 2021, Hubei province 2021, Jiangxi province 2021, Shandong province 2021, Tianjin 2021, Guizhou province 2020, Heilongjiang province 2020, Gansu province 2019, Guangxi Zhuang Autonomous Region 2019, Guizhou province 2019, Guangxi Zhuang Autonomous Region 2018, Yunnan province 2018 | Antineoplastic and immunomodulating agents | 13 | YES |
| 29 | Ketamine | Injection | Hubei province 2021, Shanxi Province 2021, Tianjin 2021, Xinjiang Uygur Autonomous Region 2021, Jilin province 2020, Liaoning province 2020, Shanxi Province 2020, Shaanxi province 2020, Tianjin 2019, Guizhou province 2019, Guangxi Zhuang Autonomous Region 2019, Shaanxi province 2019, Hunan province 2018 | Nervous system | 13 | NO |
| 30 | Bleomycin A5 | Injection | Beijing 2020, Guangxi Zhuang Autonomous Region 2021, Hainan province 2021, Hubei province 2021, Jiangxi province 2021, Tianjin 2021, Shandong province 2021, Shanghai 2021, Guizhou province 2020, Gansu province 2019, Guangdong province 2019, Guangxi Zhuang Autonomous Region 2019, Guizhou province 2019 | Antineoplastic and immunomodulating agents | 13 | YES |
| 31 | Phenobarbital | Injection | Beijing 2020, Hubei province 2021, Tianjin 2021, Shandong province 2021, Jilin province 2020, Jiangsu province 2020, Shanxi Province 2020, Guangxi Zhuang Autonomous Region 2019, Tianjin 2019, Yunnan province 2018, Shanghai 2018, Inner Mongolia Autonomous Region 2018 | Nervous system | 12 | YES |
| 32 | Sodium Thiosulfate | Injection | Beijing 2020, Guangxi Zhuang Autonomous Region 2021, Hainan province 2021, Hubei province 2021, Shandong province 2021, Shanghai 2021, Tianjin 2021, Xinjiang Uygur Autonomous Region 2021, Hainan province 2020, Guangdong province 2019, Guangxi Zhuang Autonomous Region 2019, Shanghai 2019 | Various | 12 | YES |
| 33 | Oxytocin | Injection | Beijing 2020, Hubei province 2021, Tianjin 2021, Xinjiang Uygur Autonomous Region 2021, Shanghai 2020, Shanghai 2019, Gansu province 2018, Hebei province 2018, Inner Mongolia Autonomous Region 2018, Ningxia Hui Autonomous Region 2018, Shanghai 2018, Yunnan province 2018 | Systematic hormonal preparations, sex hormones and insulins | 12 | YES |
| 34 | Verapamil | Injection | Beijing 2020, Hubei province 2021, Liaoning province 2021, Tianjin 2021, Xinjiang Uygur Autonomous Region 2021, Jiangsu province 2020, Ningxia Hui Autonomous Region 2020, Shanxi Province 2020, Gansu province 2019, Tianjin 2019, Gansu province 2018, Shanghai 2018 | Cardiovascular system | 12 | YES |
| 35 | Metaraminol | Injection | Guangxi Zhuang Autonomous Region 2021, Jiangxi province 2021, Shandong province 2021, Hubei province 2021, Xinjiang Uygur Autonomous Region 2021, Shanxi Province 2020, Gansu province 2019, Guangdong province 2019, Guangxi Zhuang Autonomous Region 2019, Hunan province 2018, Inner Mongolia Autonomous Region 2018, Ningxia Hui Autonomous Region 2018 | Cardiovascular system | 12 | NO |
| 36 | Adrenocorticotropine | Injection | Beijing 2020, Guangxi Zhuang Autonomous Region 2021, Hubei province 2021, Jiangxi province 2021, Shanxi Province 2021, Tianjin 2021, Shanxi Province 2020, Gansu province 2019, Guangxi Zhuang Autonomous Region 2019, Shaanxi province 2019, Shanghai 2018 | Systematic hormonal preparations, sex hormones and insulins | 11 | YES |
| 37 | Diazepam | Injection | Beijing 2020, Hubei province 2021, Jiangsu province 2021, Tianjin 2021, Shanghai 2021, Jiangsu province 2020, Shanghai 2020, Guangdong province 2019, Guangxi Zhuang Autonomous Region 2019, Tianjin 2019, Inner Mongolia Autonomous Region 2018 | Nervous system | 11 | YES |
| 38 | Dopamine | Injection | Beijing 2020, Hubei province 2021, Tianjin 2021, Xinjiang Uygur Autonomous Region 2021, Jiangsu province 2020, Gansu province 2019, Guangxi Zhuang Autonomous Region 2019, Shanghai 2019, Gansu province 2018, Inner Mongolia Autonomous Region 2018, Yunnan province 2018 | Nervous system | 11 | YES |
| 39 | Furosemide | Injection | Beijing 2020, Hubei province 2021, Jiangsu province 2021, Tianjin 2021, Xinjiang Uygur Autonomous Region 2021, Shanghai 2021, Shanghai 2020, Guangdong province 2019, Gansu province 2018, Hebei province 2018, Yunnan province 2018 | Cardiovascular system | 11 | YES |
| 40 | Magnesium Sulfate | Injection | Beijing 2020, Hubei province 2021, Jiangsu province 2021, Tianjin 2021, Xinjiang Uygur Autonomous Region 2021, Shanghai 2019, Gansu province 2018, Guangxi Zhuang Autonomous Region 2018, Yunnan province 2018, Shanghai 2018, Inner Mongolia Autonomous Region 2018 | Nervous system | 11 | YES |
| 41 | Vincristine | Injection | Beijing 2020, Guangxi Zhuang Autonomous Region 2021, Hubei province 2021, Jiangxi province 2021, Tianjin 2021, Heilongjiang province 2020, Gansu province 2019, Guangxi Zhuang Autonomous Region 2019, Guizhou province 2019, Shanghai 2019, Hunan province 2018 | Antineoplastic and immunomodulating agents | 11 | YES |
| 42 | Thiamazole | Tablet | Beijing 2020, Hubei province 2021, Guangxi Zhuang Autonomous Region 2021, Tianjin 2021, Liaoning province 2020, Guizhou province 2020, Guangxi Zhuang Autonomous Region 2019, Guizhou province 2019, Shanghai 2021, Shanghai 2018 | Systematic hormonal preparations, sex hormones and insulins | 10 | YES |
| 43 | Arginine | Injection | Beijing 2020, Guangxi Zhuang Autonomous Region 2021, Hainan province 2021, Hubei province 2021, Tianjin 2021, Shanghai 2021, Guangxi Zhuang Autonomous Region 2019, Gansu province 2018, Guangxi Zhuang Autonomous Region 2018, Inner Mongolia Autonomous Region 2018 | Blood and blood forming organs | 10 | YES |
| 44 | Acetamide | Injection | Beijing 2020, Hainan province 2021, Hubei province 2021, Jiangxi province 2021, Liaoning province 2021, Shandong province 2021, Tianjin 2021, Ningxia Hui Autonomous Region 2020, Gansu province 2018, Ningxia Hui Autonomous Region 2018 | Various | 10 | YES |
| 45 | Oryzanol | Tablet | Shanghai 2021, Hunan province 2020, Shanghai 2020, Gansu province 2019, Shanghai 2019, Gansu province 2018, Guangxi Zhuang Autonomous Region 2018, Hunan province 2018, Shanghai 2018, Yunnan province 2018 | Nervous system | 10 | NO |
| 46 | Tetanus antitoxin | Injection | Guizhou province 2021, Hubei province 2021, Jiangsu province 2021, Hainan province 2020, Jiangsu province 2020, Guangxi Zhuang Autonomous Region 2019, Guangdong province 2019, Gansu province 2018, Inner Mongolia Autonomous Region 2018, Shanghai 2018 | Anti-infectives for systemic use | 10 | NO |
| 47 | Hydroxycarbamide | Tablet | Jiangxi province 2021, Hubei province 2021, Shandong province 2021, Hunan province 2020, Heilongjiang province 2020, Jilin province 2020, Guangxi Zhuang Autonomous Region 2019, Guangxi Zhuang Autonomous Region 2018, Hunan province 2018, Inner Mongolia Autonomous Region 2018 | Antineoplastic and immunomodulating agents | 10 | NO |
| 48 | Cyclophosphamide | Injection | Beijing 2020, Guangxi Zhuang Autonomous Region 2021, Guangxi Zhuang Autonomous Region 2021, Hubei province 2021, Jiangxi province 2021, Tianjin 2021, Shanxi Province 2020, Guangxi Zhuang Autonomous Region 2019, Gansu province 2018 | Antineoplastic and immunomodulating agents | 9 | YES |
| 49 | Methylene Blue | Injection | Beijing 2020, Hubei province 2021, Shandong province 2021, Tianjin 2021, Ningxia Hui Autonomous Region 2020, Gansu province 2019, Guangdong province 2019, Inner Mongolia Autonomous Region 2018, Ningxia Hui Autonomous Region 2018 | Various | 9 | YES |
| 50 | Pralidoxime Iodide | Injection | Guangxi Zhuang Autonomous Region 2021, Jiangsu province 2021, Shandong province 2021, Gansu province 2019, Guangdong province 2019, Guangxi Zhuang Autonomous Region 2019, Ningxia Hui Autonomous Region 2018, Yunnan province 2018, Inner Mongolia Autonomous Region 2018 | Various | 9 | NO |
| 51 | Chlorpromazine | Injection | Guangxi Zhuang Autonomous Region 2021, Sichuan province 2021, Hainan province 2020, Shanxi Province 2020, Guangxi Zhuang Autonomous Region 2019, Gansu province 2019, Gansu province 2018, Inner Mongolia Autonomous Region 2018, Yunnan province 2018 | Nervous system | 9 | NO |
| 52 | Phenylephrine | Injection | Guangxi Zhuang Autonomous Region 2021, Xinjiang Uygur Autonomous Region 2021, Ningxia Hui Autonomous Region 2020, Gansu province 2019, Guangxi Zhuang Autonomous Region 2019, Gansu province 2018, Guangxi Zhuang Autonomous Region 2018, Ningxia Hui Autonomous Region 2018, Yunnan province 2018 | Cardiovascular system | 9 | NO |
| 53 | Nystatin | Tablet | Guangxi Zhuang Autonomous Region 2021, Tianjin 2021, Guangxi Zhuang Autonomous Region 2019, Shanghai 2019, Tianjin 2019, Hebei province 2018, Guangxi Zhuang Autonomous Region 2018, Shanghai 2018, Yunnan province 2018 | Dermatologicals | 9 | NO |
| 54 | Sodium Dimercaptopropane Sulfonate | Injection | Beijing 2020, Guizhou province 2021, Hubei province 2021, Shandong province 2021, Tianjin 2021, Jiangsu province 2020, Guangxi Zhuang Autonomous Region 2019, Shanghai 2019 | Various | 8 | YES |
| 55 | Hydrocortisone | Injection | Beijing 2020, Hainan province 2021, Hubei province 2021, Tianjin 2021, Xinjiang Uygur Autonomous Region 2021, Hainan province 2020, Gansu province 2018, Inner Mongolia Autonomous Region 2018 | Systematic hormonal preparations, sex hormones and insulins | 8 | YES |
| 56 | Mercaptopurine | Tablet | Beijing 2020, Guangxi Zhuang Autonomous Region 2021, Hubei province 2021, Jiangxi province 2021, Tianjin 2021, Guangxi Zhuang Autonomous Region 2019, Guizhou province 2019, Shanghai 2018 | Antineoplastic and immunomodulating agents | 8 | YES |
| 57 | Iodinated Oil | Injection | Guangxi Zhuang Autonomous Region 2021, Jilin province 2020, Guangxi Zhuang Autonomous Region 2019, Gansu province 2019, Shanghai 2019, Guangxi Zhuang Autonomous Region 2018, Gansu province 2018, Ningxia Hui Autonomous Region 2018 | Alimentary tract and metabolism | 8 | NO |
| 58 | Scopolamine | Injection | Shanghai 2021, Jiangsu province 2021, Jiangsu province 2020, Ningxia Hui Autonomous Region 2020, Guangxi Zhuang Autonomous Region 2019, Guizhou province 2019, Inner Mongolia Autonomous Region 2018, Yunnan province 2018 | Alimentary tract and metabolism | 8 | NO |
| 59 | Promethazine | Injection | Guangxi Zhuang Autonomous Region 2021, Sichuan province 2021, Xinjiang Uygur Autonomous Region 2021, Hunan province 2020, Jiangsu province 2020, Guangxi Zhuang Autonomous Region 2019, Gansu province 2018, Inner Mongolia Autonomous Region 2018 | Respiratory system | 8 | NO |
| 60 | Penicillamine | Tablet | Beijing 2020, Hubei province 2021, Jiangxi province 2021, Tianjin 2021, Guangdong province 2019, Guangxi Zhuang Autonomous Region 2019, Hunan province 2018 | Musculo-skeletal system | 7 | YES |
| 61 | Amiodarone | Injection | Beijing 2020, Guangxi Zhuang Autonomous Region 2021, Hubei province 2021, Jiangsu province 2021, Tianjin 2021, Guangdong province 2019, Guangxi Zhuang Autonomous Region 2019 | Cardiovascular system | 7 | YES |
| 62 | Digoxin | Oral liquid | Beijing 2020, Guangxi Zhuang Autonomous Region 2021, Hainan province 2021, Jiangxi province 2021, Hubei province 2021, Jiangsu province 2021, Tianjin 2021 | Cardiovascular system | 7 | YES |
| 63 | Etoposide | Injection | Beijing 2020, Hubei province 2021, Shanxi Province 2021, Shanxi Province 2020, Shaanxi province 2020, Guizhou province 2019, Shaanxi province 2019 | Antineoplastic and immunomodulating agents | 7 | YES |
| 64 | Haloperidol | Injection | Jiangsu province 2021, Gansu province 2019, Guizhou province 2019, Shanghai 2019, Guangxi Zhuang Autonomous Region 2018, Shanghai 2018, Inner Mongolia Autonomous Region 2018 | Nervous system | 7 | NO |
| 65 | Heparin | Injection | Jiangsu province 2021, Tianjin 2021, Shanghai 2021, Ningxia Hui Autonomous Region 2020, Shanghai 2020, Gansu province 2019, Tianjin 2019 | Blood and blood forming organs | 7 | NO |
| 66 | Suxamethonium Chloride | Injection | Shandong province 2021, Hubei province 2021, Jiangsu province 2020, Gansu province 2019, Shanghai 2019, Shanghai 2018, Inner Mongolia Autonomous Region 2018 | Musculo-skeletal system | 7 | NO |
| 67 | Chymotrypsin | Injection | Guizhou province 2021, Jilin province 2020, Ningxia Hui Autonomous Region 2020, Guangxi Zhuang Autonomous Region 2019, Guangxi Zhuang Autonomous Region 2018, Ningxia Hui Autonomous Region 2018, Shanghai 2018 | Sensory organs | 7 | NO |
| 68 | Chorionic Gonadotrophin | Injection | Guangxi Zhuang Autonomous Region 2021, Jiangsu province 2021, Gansu province 2019, Guangxi Zhuang Autonomous Region 2019, Shanghai 2019, Gansu province 2018, Guangxi Zhuang Autonomous Region 2018 | Genito urinary system and sex hormones | 7 | NO |
| 69 | Sodium Bicarbonate | Tablet | Jiangsu province 2021, Xinjiang Uygur Autonomous Region 2021, Jilin province 2020, Gansu province 2018, Hebei province 2018, Inner Mongolia Autonomous Region 2018, Yunnan province 2018 | Alimentary tract and metabolism | 7 | NO |
| 70 | Clofazimine | Capsule | Beijing 2020, Hubei province 2021, Jiangxi province 2021, Tianjin 2021, Guizhou province 2020, Shanghai 2019 | Anti-infectives for systemic use | 6 | YES |
| 71 | Thrombin | Lyophilizing Powder | Beijing 2020, Hubei province 2021, Liaoning province 2021, Tianjin 2021, Hunan province 2020, Guizhou province 2019 | Blood and blood forming organs | 6 | YES |
| 72 | Sodium Nitroprusside | Injection | Beijing 2020, Hubei province 2021, Jiangsu province 2021, Tianjin 2021, Gansu province 2018, Inner Mongolia Autonomous Region 2018 | Cardiovascular system | 6 | YES |
| 73 | Aminophylline | Injection | Xinjiang Uygur Autonomous Region 2021, Shanghai 2021, Shanghai 2020, Gansu province 2018, Yunnan province 2018, Inner Mongolia Autonomous Region 2018 | Respiratory system | 6 | NO |
| 74 | Bupivacaine | Injection | Hainan province 2021, Hainan province 2020, Ningxia Hui Autonomous Region 2020, Gansu province 2018, Hunan province 2018, Ningxia Hui Autonomous Region 2018 | Nervous system | 6 | NO |
| 75 | Estriol | Cream | Hubei province 2021, Liaoning province 2021, Sichuan province 2021, Guizhou province 2019, Hunan province 2018, Yunnan province 2018 | Genito urinary system and sex hormones | 6 | NO |
| 76 | Etamsylate | Injection | Jiangsu province 2021, Xinjiang Uygur Autonomous Region 2021, Shanxi Province 2020, Gansu province 2018, Inner Mongolia Autonomous Region 2018, Yunnan province 2018 | Blood and blood forming organs | 6 | NO |
| 77 | Metoclopramide | Injection | Jiangsu province 2021, Xinjiang Uygur Autonomous Region 2021, Guangxi Zhuang Autonomous Region 2018, Ningxia Hui Autonomous Region 2018, Shanxi Province 2020, Gansu province 2019 | Alimentary tract and metabolism | 6 | NO |
| 78 | Labetalol | Injection | Jiangsu province 2021, Hunan province 2020, Shaanxi province 2020, Shaanxi province 2019, Hunan province 2018, Yunnan province 2018 | Cardiovascular system | 6 | NO |
| 79 | Propranolol | Tablet | Jilin province 2020, Shanghai 2020, Shanghai 2019, Gansu province 2018, Inner Mongolia Autonomous Region 2018, Yunnan province 2018 | Cardiovascular system | 6 | NO |
| 80 | Human albumin | Injection | Hainan province 2021, Shanghai 2021, Hainan province 2020, Shanghai 2020, Shanghai 2019, Shanghai 2018 | Blood and blood forming organs | 6 | NO |
| 81 | Isoniazid | Injection | Shanghai 2020, Gansu province 2019, Shanghai 2019, Guangxi Zhuang Autonomous Region 2018, Ningxia Hui Autonomous Region 2018, Yunnan province 2018 | Anti-infectives for systemic use | 6 | NO |
| 82 | Testosterone Undecanoate | Capsule | Guizhou province 2021, Jiangsu province 2021, Shanghai 2021, Heilongjiang province 2020, Jilin province 2020, Guizhou province 2019 | Genito urinary system and sex hormones | 6 | NO |
| 83 | Stanozolol | Tablet | Guangxi Zhuang Autonomous Region 2021, Jiangxi province 2021, Shandong province 2021, Tianjin 2021, Guangxi Zhuang Autonomous Region 2019, Tianjin 2019 | Alimentary tract and metabolism | 6 | NO |
| 84 | Aminophylline | Tablet | Jiangsu province 2021, Liaoning province 2020, Hebei province 2018, Gansu province 2018, Yunnan province 2018 | Respiratory system | 5 | NO |
| 85 | Testosterone Propionate | Injection | Liaoning province 2020, Gansu province 2019, Guangxi Zhuang Autonomous Region 2018, Ningxia Hui Autonomous Region 2018, Yunnan province 2018 | Genito urinary system and sex hormones | 5 | NO |
| 86 | Progesterone | Injection | Xinjiang Uygur Autonomous Region 2021, Gansu province 2018, Hebei province 2018, Inner Mongolia Autonomous Region 2018, Yunnan province 2018 | Genito urinary system and sex hormones | 5 | NO |
| 87 | Metoclopramide | Tablet | Jiangsu province 2021, Gansu province 2019, Guangxi Zhuang Autonomous Region 2018, Hunan province 2018, Yunnan province 2018 | Alimentary tract and metabolism | 5 | NO |
| 88 | Carbidopa and Levodopa | Controlled/Sustained-release Tablet | Liaoning province 2021, Tianjin 2021, Shandong province 2021, Shaanxi province 2020, Shaanxi province 2019 | Nervous system | 5 | NO |
| 89 | Rifapentine | Capsule | Shanghai 2020, Shaanxi province 2020, Gansu province 2018, Shanghai 2018, Inner Mongolia Autonomous Region 2018 | Anti-infectives for systemic use | 5 | NO |
| 90 | Procaine | Injection | Hainan province 2020, Guangxi Zhuang Autonomous Region 2019, Gansu province 2018, Guangxi Zhuang Autonomous Region 2018, Ningxia Hui Autonomous Region 2018 | Nervous system | 5 | NO |
| 91 | Anisodamine | Tablet | Jiangsu province 2021, Xinjiang Uygur Autonomous Region 2021, Ningxia Hui Autonomous Region 2020, Ningxia Hui Autonomous Region 2018, Yunnan province 2018 | Alimentary tract and metabolism | 5 | NO |
| 92 | Folic acid | Tablet | Shanghai 2021, Shanghai 2020, Gansu province 2018, Hebei province 2018, Shanghai 2018 | Blood and blood forming organs | 5 | NO |
| 93 | Dextran 40 Sodium Chloride | Injection | Jiangsu province 2020, Shanxi Province 2020, Gansu province 2019, Guangdong province 2019, Inner Mongolia Autonomous Region 2018 | Blood and blood forming organs | 5 | NO |
| 94 | Aminomethylbenzoic Acid | Injection | Hubei province 2021, Hunan province 2020, Heilongjiang province 2020, Shanxi Province 2020, Gansu province 2019 | Blood and blood forming organs | 5 | NO |
| 95 | Menadione Sodium Bisulfite | Injection | Xinjiang Uygur Autonomous Region 2021, Guizhou province 2020, Heilongjiang province 2020, Gansu province 2019, Gansu province 2018 | Blood and blood forming organs | 5 | NO |
| 96 | Snake Antivenins | Injection | Beijing 2020, Hubei province 2021, Tianjin 2021, Guangdong province 2019 | Anti-infectives for systemic use | 4 | YES |
| 97 | Naloxone | Injection | Beijing 2020, Guizhou province 2021, Hubei province 2021, Tianjin 2021 | Nervous system | 4 | YES |
| 98 | Diphenhydramine | Injection | Guangdong province 2019, Gansu province 2018, Guangxi Zhuang Autonomous Region 2018, Ningxia Hui Autonomous Region 2018 | Dermatologicals | 4 | NO |
| 99 | Doxepin | Tablet | Guangxi Zhuang Autonomous Region 2019, Hebei province 2018, Guangxi Zhuang Autonomous Region 2018, Inner Mongolia Autonomous Region 2018 | Nervous system | 4 | NO |
| 100 | Droperidol | Injection | Yunnan province 2018, Gansu province 2019, Guangxi Zhuang Autonomous Region 2018, Inner Mongolia Autonomous Region 2018 | Nervous system | 4 | NO |
| 101 | Compound Aminophenazone and Barbital | Injection | Jilin province 2020, Shanxi Province 2020, Gansu province 2018, Ningxia Hui Autonomous Region 2018 | Nervous system | 4 | NO |
| 102 | Homoharringtonine | Injection | Guangxi Zhuang Autonomous Region 2021, Guangxi Zhuang Autonomous Region 2019, Gansu province 2019, Shanghai 2019 | Antineoplastic and immunomodulating agents | 4 | NO |
| 103 | Metronidazole | Tablet | Jiangsu province 2021, Gansu province 2018, Hebei province 2018, Hunan province 2018 | Antiparasitic products, insecticides and repellents | 4 | NO |
| 104 | Chloramphenicol | Injection | Guangxi Zhuang Autonomous Region 2021, Hainan province 2021, Hainan province 2020, Guangxi Zhuang Autonomous Region 2019 | Anti-infectives for systemic use | 4 | NO |
| 105 | Human immunoglobulin (ph4) | Injection | Shanghai 2021, Shanghai 2020, Shanghai 2019, Shanghai 2018 | Anti-infectives for systemic use | 4 | NO |
| 106 | Human Prothrombin Complex | Injection | Guangxi Zhuang Autonomous Region 2021, Shanghai 2021, Shanghai 2020, Guangxi Zhuang Autonomous Region 2019 | Blood and blood forming organs | 4 | NO |
| 107 | Sodium Lactate Ringer's Injection | Injection | Guangxi Zhuang Autonomous Region 2021, Hainan province 2021, Hainan province 2020, Guangxi Zhuang Autonomous Region 2019 | Blood and blood forming organs | 4 | NO |
| 108 | Tamoxifen | Tablet | Guizhou province 2021, Shanghai 2021, Shaanxi province 2020, Shanghai 2019 | Antineoplastic and immunomodulating agents | 4 | NO |
| 109 | Verapamil | Tablet | Guangxi Zhuang Autonomous Region 2021, Jiangsu province 2021, Ningxia Hui Autonomous Region 2020, Ningxia Hui Autonomous Region 2018 | Cardiovascular system | 4 | NO |
| 110 | Mannitol | Injection | Beijing 2020, Hubei province 2021, Tianjin 2021 | Blood and blood forming organs | 3 | YES |
| 111 | Tretinoin | Tablet | Beijing 2020, Hubei province 2021, Hunan province 2018 | Dermatologicals | 3 | YES |
| 112 | Tranexamic acid | Tablet | Jiangsu province 2021, Hunan province 2020, Shaanxi province 2020 | Blood and blood forming organs | 3 | NO |
| 113 | Praziquantel | Tablet | Guangxi Zhuang Autonomous Region 2021, Liaoning province 2020, Gansu province 2018 | Antiparasitic products, insecticides and repellents | 3 | NO |
| 114 | Digoxin | Tablet | Guangxi Zhuang Autonomous Region 2021, Sichuan province 2021, Xinjiang Uygur Autonomous Region 2021 | Cardiovascular system | 3 | NO |
| 115 | Dexamethasone | Tablet | Jiangsu province 2021, Ningxia Hui Autonomous Region 2018, Hebei province 2018 | Systematic hormonal preparations, sex hormones and insulins | 3 | NO |
| 116 | Polymyxin B | Injection | Guangxi Zhuang Autonomous Region 2021, Guangxi Zhuang Autonomous Region 2019, Guizhou province 2019 | Anti-infectives for systemic use | 3 | NO |
| 117 | Dactinomycin | Injection | Gansu province 2019, Guizhou province 2019, Shanghai 2018 | Antineoplastic and immunomodulating agents | 3 | NO |
| 118 | Fluorouracil | Injection | Shanghai 2020, Gansu province 2019, Shanghai 2018 | Antineoplastic and immunomodulating agents | 3 | NO |
| 119 | Tropicamide | Eye Drops | Hubei province 2021, Gansu province 2018, Hunan province 2018 | Sensory organs | 3 | NO |
| 120 | Erythromycin | Eye ointment | Xinjiang Uygur Autonomous Region 2021, Gansu province 2018, Hebei province 2018 | Sensory organs | 3 | NO |
| 121 | Inosine | Injection | Guangxi Zhuang Autonomous Region 2021, Xinjiang Uygur Autonomous Region 2021, Gansu province 2018 | Blood and blood forming organs | 3 | NO |
| 122 | Methyltestosterone | Tablet | Ningxia Hui Autonomous Region 2020, Ningxia Hui Autonomous Region 2018, Hunan province 2018 | Genito urinary system and sex hormones | 3 | NO |
| 123 | Desferrioxamine | Injection | Guangxi Zhuang Autonomous Region 2021, Guangxi Zhuang Autonomous Region 2019, Gansu province 2018 | Various | 3 | NO |
| 124 | Carbamazepine | Tablet | Gansu province 2019, Gansu province 2018, Yunnan province 2018 | Nervous system | 3 | NO |
| 125 | Carboprost Methylate | Suppository | Shanghai 2021, Hunan province 2020, Jilin province 2020 | Genito urinary system and sex hormones | 3 | NO |
| 126 | Agkistrodon Halys Antivenin/Snake Antivenins | Injection | Jiangsu province 2020, Liaoning province 2020, Shanghai 2018 | Anti-infectives for systemic use | 3 | NO |
| 127 | Amphotericin B | Injection | Liaoning province 2020, Gansu province 2019, Guangdong province 2019 | Anti-infectives for systemic use | 3 | NO |
| 128 | Fosfomycin | Injection | Jiangsu province 2021, Liaoning province 2021, Jilin province 2020 | Anti-infectives for systemic use | 3 | NO |
| 129 | Rotundine | Injection | Hunan province 2020, Guangxi Zhuang Autonomous Region 2019, Guangxi Zhuang Autonomous Region 2018 | Nervous system | 3 | NO |
| 130 | Potassium Aspartate and Magnesium Aspartate | Injection | Xinjiang Uygur Autonomous Region 2021, Hainan province 2020, Gansu province 2019 | Alimentary tract and metabolism | 3 | NO |
| 131 | Human Tetanus Immunoglobulin | Injection | Shanghai 2021, Shanghai 2019, Shanghai 2018 | Anti-infectives for systemic use | 3 | NO |
| 132 | Propafenone | Tablet | Tianjin 2021, Tianjin 2019, Hebei province 2018 | Cardiovascular system | 3 | NO |
| 133 | Norethindrone | Tablet | Gansu province 2018, Inner Mongolia Autonomous Region 2018, Yunnan province 2018 | Genito urinary system and sex hormones | 3 | NO |
| 134 | (Human) Coagulation Factor Ⅷ | Injection | Shanghai 2021, Sichuan province 2021, Shanghai 2020 | Blood and blood forming organs | 3 | NO |
| 135 | human fibrinogen | Injection | Shanghai 2020, Shanghai 2019, Shanghai 2018 | Blood and blood forming organs | 3 | NO |
| 136 | Erythromycin lactobionate | Injection | Jiangsu province 2020, Gansu province 2019, Gansu province 2018 | Anti-infectives for systemic use | 3 | NO |
| 137 | Carbazochrome | Tablet | Guangxi Zhuang Autonomous Region 2021, Guangxi Zhuang Autonomous Region 2018, Gansu province 2018 | Blood and blood forming organs | 3 | NO |
| 138 | Lithium Carbonate | Tablet | Guangxi Zhuang Autonomous Region 2021, Guangxi Zhuang Autonomous Region 2019, Gansu province 2018 | Nervous system | 3 | NO |
| 139 | Tibolone | Tablet | Guangxi Zhuang Autonomous Region 2021, Guizhou province 2020, Guangxi Zhuang Autonomous Region 2019 | Genito urinary system and sex hormones | 3 | NO |
| 140 | Tropicamide | Eye Drops | Guizhou province 2019, Guangxi Zhuang Autonomous Region 2018, Hebei province 2018 | Sensory organs | 3 | NO |
| 141 | Raceanisodamine | Tablet | Hebei province 2018, Inner Mongolia Autonomous Region 2018, Yunnan province 2018 | Alimentary tract and metabolism | 3 | NO |
| 142 | Raceanisodamine | Injection | Hebei province 2018, Gansu province 2018, Inner Mongolia Autonomous Region 2018 | Alimentary tract and metabolism | 3 | NO |
| 143 | Ifosfamide | Injection | Guangxi Zhuang Autonomous Region 2021, Gansu province 2019, Hunan province 2018 | Antineoplastic and immunomodulating agents | 3 | NO |
| 144 | Papaverine | Injection | Gansu province 2019, Guangxi Zhuang Autonomous Region 2019, Gansu province 2018 | Genito urinary system and sex hormones | 3 | NO |
| 145 | Dextran 40 and glucose injection | Injection | Hainan province 2021, Hunan province 2018, Shanghai 2018 | Blood and blood forming organs | 3 | NO |
| 146 | Primacaine Adrenaline | Injection | Guangxi Zhuang Autonomous Region 2018, Gansu province 2018 | Nervous system | 2 | NO |
| 147 | Atenolol | Tablet | Jiangsu province 2021, Gansu province 2018 | Cardiovascular system | 2 | NO |
| 148 | Alteplase | Injection | Shaanxi province 2020, Shaanxi province 2019 | Blood and blood forming organs | 2 | NO |
| 149 | Aminocaproic Acid | Injection | Gansu province 2019, Gansu province 2018 | Blood and blood forming organs | 2 | NO |
| 150 | Tranexamic acid | Injection | Hainan province 2020, Shanxi Province 2020 | Blood and blood forming organs | 2 | NO |
| 151 | Phenobarbital Sodium | Tablet | Tianjin 2019, Yunnan province 2018 | Nervous system | 2 | NO |
| 152 | Oxacillin Sodium | Injection | Guizhou province 2021, Hainan province 2021, | Anti-infectives for systemic use | 2 | NO |
| 153 | Sodium Valproate | Tablet | Guangxi Zhuang Autonomous Region 2021Sichuan province 2021, Guangxi Zhuang Autonomous Region 2019 | Nervous system | 2 | NO |
| 154 | Dimenhydrinate Tablets | Tablet | Jiangsu province 2021, Gansu province 2018 | Respiratory system | 2 | NO |
| 155 | Theophylline | Sustained-release Capsule | Hainan province 2021, Hainan province 2020 | Respiratory system | 2 | NO |
| 156 | Sodium aminosalicylate | Tablet (enteric-coated) | Shanghai 2021, Shanghai 2018 | Anti-infectives for systemic use | 2 | NO |
| 157 | Fluphenazine | Tablet | Jiangsu province 2020, Gansu province 2018 | Nervous system | 2 | NO |
| 158 | Furazolidone | Tablet | Gansu province 2018, Yunnan province 2018 | Genito urinary system and sex hormones | 2 | NO |
| 159 | Coenzyme A | Injection | Xinjiang Uygur Autonomous Region 2021, Gansu province 2018 | Blood and blood forming organs | 2 | NO |
| 160 | Aminobutynic Acid and Vitamin E | Capsule | Shaanxi province 2020, Shaanxi province 2019 | Nervous system | 2 | NO |
| 161 | Betamethasone | Injection | Hainan province 2021, Hainan province 2020 | Systematic hormonal preparations, sex hormones and insulins | 2 | NO |
| 162 | Compound Diphenoxylate | Tablet | Gansu province 2018, Hebei province 2018 | Alimentary tract and metabolism | 2 | NO |
| 163 | Cyclophosphamide | Tablet | Tianjin 2021, Tianjin 2019 | Antineoplastic and immunomodulating agents | 2 | NO |
| 164 | Titanoreine | Suppository | Gansu province 2018, Hunan province 2018 | Dermatologicals | 2 | NO |
| 165 | Potassium Permanganate | Vaginal tablet | Guangdong province 2019, Gansu province 2019 | Various | 2 | NO |
| 166 | Gongxuening | Capsule | Gansu province 2018, Yunnan province 2018 | Genito urinary system and sex hormones | 2 | NO |
| 167 | Hydrogen peroxide | Vaginal Solution | Shanghai 2019, Shanghai 2018 | Dermatologicals | 2 | NO |
| 168 | Halcinonide | Vaginal Solution | Shanghai 2019, Shanghai 2018 | Dermatologicals | 2 | NO |
| 169 | Inosine | Tablet | Gansu province 2018, Hebei province 2018 | Alimentary tract and metabolism | 2 | NO |
| 170 | Galanthamine | Injection | Hubei province 2021, Jiangsu province 2020 | Nervous system | 2 | NO |
| 171 | Medroxyprogesterone | Tablet (dispersible) | Yunnan province 2018, Inner Mongolia Autonomous Region 2018 | Antineoplastic and immunomodulating agents | 2 | NO |
| 172 | Metronidazole and sodium chloride | Injection | Gansu province 2018, Yunnan province 2018 | Anti-infectives for systemic use | 2 | NO |
| 173 | Thyroid | Tablet | Ningxia Hui Autonomous Region 2020, Ningxia Hui Autonomous Region 2018 | Systematic hormonal preparations, sex hormones and insulins | 2 | NO |
| 174 | Carbidopaand Levodopa CR Tablets | Tablet | Jiangsu province 2021, Jiangxi province 2021 | Nervous system | 2 | NO |
| 175 | Aspirin-Dl-Lysine | Injection | Gansu province 2019, Ningxia Hui Autonomous Region 2018 | Nervous system | 2 | NO |
| 176 | Ranitidine | Capsule | Jiangsu province 2021, Hebei province 2018 | Alimentary tract and metabolism | 2 | NO |
| 177 | Lidocaine | Injection | Jiangsu province 2021, Hebei province 2018 | Cardiovascular system | 2 | NO |
| 178 | Azathioprine | Tablet | Shanxi Province 2021, Guangxi Zhuang Autonomous Region 2021 | Antineoplastic and immunomodulating agents | 2 | NO |
| 179 | Calamine | Lotion | Ningxia Hui Autonomous Region 2018, Yunnan province 2018 | Dermatologicals | 2 | NO |
| 180 | Chlorpromazine | Tablet | Gansu province 2018, Yunnan province 2018 | Nervous system | 2 | NO |
| 181 | Calcium Chloride | Injection | Jiangsu province 2021, Jiangsu province 2020 | Alimentary tract and metabolism | 2 | NO |
| 182 | Chlorquinaldol Promestriene Vaginal Tablets | Vaginal tablet | Shaanxi province 2020, Shaanxi province 2019 | Genito urinary system and sex hormones | 2 | NO |
| 183 | Clonazepam | Injection | Tianjin 2021, Tianjin 2019 | Nervous system | 2 | NO |
| 184 | Metoprolol | Injection | Gansu province 2018, Guangxi Zhuang Autonomous Region 2018 | Cardiovascular system | 2 | NO |
| 185 | Hydrocortisone | Tablet | Ningxia Hui Autonomous Region 2020, Ningxia Hui Autonomous Region 2018 | Systematic hormonal preparations, sex hormones and insulins | 2 | NO |
| 186 | Recombinant Human Follitropin | Injection | Shaanxi province 2020, Shaanxi province 2019 | Genito urinary system and sex hormones | 2 | NO |
| 187 | Vasopvessin Tannic | Injection | Shanghai 2021, Hunan province 2018 | Systematic hormonal preparations, sex hormones and insulins | 2 | NO |
| 188 | Triethanolamine | Cream | Shaanxi province 2020, Shaanxi province 2019 | Dermatologicals | 2 | NO |
| 189 | Moist exposed burn ointment（MEBO） | Cream | Jilin province 2020, Gansu province 2018 | Dermatologicals | 2 | NO |
| 190 | Dipyridamole | Tablet | Gansu province 2018, Guangxi Zhuang Autonomous Region 2018 | Blood and blood forming organs | 2 | NO |
| 191 | Semustine | Capsule | Gansu province 2018, Liaoning province 2020 | Antineoplastic and immunomodulating agents | 2 | NO |
| 192 | Sodium bicarbonate | Injection | Gansu province 2019, Hebei province 2018 | Blood and blood forming organs | 2 | NO |
| 193 | Vitamin B12 | Injection | Gansu province 2018, Hebei province 2018 | Blood and blood forming organs | 2 | NO |
| 194 | Vitamin B | Injection | Gansu province 2018, Yunnan province 2018 | Alimentary tract and metabolism | 2 | NO |
| 195 | Nifedipine | Tablet | Hebei province 2018, Yunnan province 2018 | Cardiovascular system | 2 | NO |
| 196 | Isosorbide Dinitrate | Tablet | Shanxi Province 2020, Gansu province 2018 | Cardiovascular system | 2 | NO |
| 197 | Calcium Folinate | Injection | Hunan province 2020, Hunan province 2018 | Various | 2 | NO |
| 198 | Ethacridine | Topical solution | Shanghai 2020, Shanghai 2019 | Dermatologicals | 2 | NO |
| 199 | Isoniazid | Tablet | Gansu province 2018, Yunnan province 2018 | Anti-infectives for systemic use | 2 | NO |
| 200 | Indocyanine Green | Injection | Tianjin 2021, Tianjin 2019 | Various | 2 | NO |
| 201 | Levodopa | Tablet | Hainan province 2021, Hunan province 2018 | Nervous system | 2 | NO |
| 202 | Diethylstilbestrol | Tablet | Liaoning province 2020, Yunnan province 2018 | Genito urinary system and sex hormones | 2 | NO |
| 203 | Promethazine | Tablet | Gansu province 2018, Ningxia Hui Autonomous Region 2018 | Respiratory system | 2 | NO |
| 204 | Clomifene | Capsule | Liaoning province 2021, Inner Mongolia Autonomous Region 2018 | Genito urinary system and sex hormones | 2 | NO |
| 205 | Clomifene | Tablet | Liaoning province 2021, Guizhou province 2019 | Genito urinary system and sex hormones | 2 | NO |
| 206 | Rifampicin | Capsule | Liaoning province 2021, Gansu province 2019 | Anti-infectives for systemic use | 2 | NO |
| 207 | Aclarubicin | Injection | Heilongjiang province 2020, Hunan province 2018 | Anti-infectives for systemic use | 2 | NO |
| 208 | Sodium Aminosalicylate | Injection | Heilongjiang province 2020, Shanghai 2020 | Anti-infectives for systemic use | 2 | NO |
| 209 | Vitamin D 2and Calcium Colloidal | Injection | Guangxi Zhuang Autonomous Region 2018, Yunnan province 2018 | Alimentary tract and metabolism | 2 | NO |
| 210 | Chloral hydrate | Rectal Solution | Guangxi Zhuang Autonomous Region 2019 | Nervous system | 1 | NO |
| 211 | Botulinum Toxin A | Injection | Shanghai 2021 | Musculo-skeletal system | 1 | NO |
| 212 | Albendazole | Capsule | Gansu province 2018 | Antiparasitic products, insecticides and repellents | 1 | NO |
| 213 | Albendazole | Tablet | Gansu province 2018 | Antiparasitic products, insecticides and repellents | 1 | NO |
| 214 | Amikacin | Injection | Gansu province 2018 | Anti-infectives for systemic use | 1 | NO |
| 215 | Atropine | Eye ointment | Liaoning province 2020 | Sensory organs | 1 | NO |
| 216 | Aciclovir | Tablet | Hebei province 2018 | Anti-infectives for systemic use | 1 | NO |
| 217 | Esmolol | Injection | Gansu province 2018 | Cardiovascular system | 1 | NO |
| 218 | Triamterene | Tablet | Liaoning province 2020 | Cardiovascular system | 1 | NO |
| 219 | Paracetamol and Codeine Phosphate Tablets(Ⅰ) | Tablet | Gansu province 2018 | Nervous system | 1 | NO |
| 220 | Aminocaproic Acid | Injection | Gansu province 2019 | Blood and blood forming organs | 1 | NO |
| 221 | Aminomethylbenzoic Acid | Tablet | Hunan province 2018 | Blood and blood forming organs | 1 | NO |
| 222 | Aminobutyric Acid | Tablet | Hunan province 2018 | Nervous system | 1 | NO |
| 223 | Ambroxol | Tablet | Hebei province 2018 | Respiratory system | 1 | NO |
| 224 | Oxybuprocaine | Eye Drops | Gansu province 2018 | Sensory organs | 1 | NO |
| 225 | Busulfan | Tablet | Liaoning province 2020 | Antineoplastic and immunomodulating agents | 1 | NO |
| 226 | Citicoline | Injection | Inner Mongolia Autonomous Region 2018 | Nervous system | 1 | NO |
| 227 | Baohe Pills | Pill | Gansu province 2018 | Alimentary tract and metabolism | 1 | NO |
| 228 | Chlorambucil | Tablet | Hunan province 2018 | Antineoplastic and immunomodulating agents | 1 | NO |
| 229 | Trihexyphenidyl | Tablet | Gansu province 2019 | Nervous system | 1 | NO |
| 230 | Phenytoin Sodium | Injection | Gansu province 2018 | Nervous system | 1 | NO |
| 231 | Pipemidic Acid | Tablet | Gansu province 2018 | Anti-infectives for systemic use | 1 | NO |
| 232 | Pyrazinamide | Capsule | Gansu province 2018 | Anti-infectives for systemic use | 1 | NO |
| 233 | Pyrazinamide | Tablet | Gansu province 2019 | Anti-infectives for systemic use | 1 | NO |
| 234 | Propylthiouracil | Tablet | Ningxia Hui Autonomous Region 2018 | Systematic hormonal preparations, sex hormones and insulins | 1 | NO |
| 235 | Protionamide | Tablet | Liaoning province 2021 | Anti-infectives for systemic use | 1 | NO |
| 236 | Protionamide | Tablet (enteric-coated) | Liaoning province 2021 | Anti-infectives for systemic use | 1 | NO |
| 237 | Imipramine | Tablet | Gansu province 2018 | Nervous system | 1 | NO |
| 238 | Hyaluronidase | Injection | Gansu province 2018 | Blood and blood forming organs | 1 | NO |
| 239 | Primaquine | Tablet | Liaoning province 2020 | Antiparasitic products, insecticides and repellents | 1 | NO |
| 240 | Budesonide | inhalation | Gansu province 2018 | Respiratory system | 1 | NO |
| 241 | Bumetanide | Tablet | Yunnan province 2021 | Cardiovascular system | 1 | NO |
| 242 | Recombinant Follitropin Beta | Injection | Shaanxi province 2019 | Genito urinary system and sex hormones | 1 | NO |
| 243 | Methazolamide | Tablet | Hainan province 2021 | Sensory organs | 1 | NO |
| 244 | Dexamethasone Acetate | Tablet | Hebei province 2018 | Systematic hormonal preparations, sex hormones and insulins | 1 | NO |
| 245 | Dexamethasone Acetate | Cream | Gansu province 2018 | Dermatologicals | 1 | NO |
| 246 | Fluocinonide | Cream | Inner Mongolia Autonomous Region 2018 | Dermatologicals | 1 | NO |
| 247 | Digoxin | Injection | Shanghai 2020 | Cardiovascular system | 1 | NO |
| 248 | Diazepam | Tablet | Hunan province 2018 | Nervous system | 1 | NO |
| 249 | Hydrocortisone Butyrate | Cream | Gansu province 2018 | Dermatologicals | 1 | NO |
| 250 | Multienzyme | Tablet | Gansu province 2018 | Alimentary tract and metabolism | 1 | NO |
| 251 | Donepezil | Tablet | Gansu province 2018 | Nervous system | 1 | NO |
| 252 | Phacolysin | Eye Drops | Gansu province 2018 | Sensory organs | 1 | NO |
| 253 | Fenofibrate | Tablet | Hunan province 2018 | Cardiovascular system | 1 | NO |
| 254 | Paracetamol,Aminophenazone,Caffeine and Chlorphenamine Maleate Tablets | Tablet | Yunnan province 2018 | Nervous system | 1 | NO |
| 255 | Phenolphthalein | Tablet | Guangxi Zhuang Autonomous Region 2018 | Alimentary tract and metabolism | 1 | NO |
| 256 | Ephedrine Hydrichloride And Nitrofurazone | Nasal Drops | Gansu province 2018 | Respiratory system | 1 | NO |
| 257 | Furosemide | Tablet | Hebei province 2018 | Cardiovascular system | 1 | NO |
| 258 | Flucytosine | Tablet | Gansu province 2018 | Anti-infectives for systemic use | 1 | NO |
| 259 | Haloperidol | Tablet | Tianjin 2021 | Nervous system | 1 | NO |
| 260 | Compound Dexamethasone Acetate Cream | Cream | Gansu province 2018 | Dermatologicals | 1 | NO |
| 261 | Compound Soudiom Acetion | Injection | Gansu province 2018 | Blood and blood forming organs | 1 | NO |
| 262 | Compound Sulfamethoxazole | Tablet | Yunnan province 2018 | Anti-infectives for systemic use | 1 | NO |
| 263 | Compound Sulfamethoxazole | Injection | Shanghai 2020 | Anti-infectives for systemic use | 1 | NO |
| 264 | Compound Sulfadiazine Zinc | Vaginal Gel | Gansu province 2018 | Dermatologicals | 1 | NO |
| 265 | Compound Flavescent Sophora and Salicylic Acid | Topical Powder | Shanghai 2019 | Dermatologicals | 1 | NO |
| 266 | Compound Vitamin | Tablet | Gansu province 2018 | Alimentary tract and metabolism | 1 | NO |
| 267 | Licorzine Granules | Granule | Hunan province 2018 | Alimentary tract and metabolism | 1 | NO |
| 268 | Heparin Calcium | Injection | Shanghai 2021 | Blood and blood forming organs | 1 | NO |
| 269 | Gou-pi Plaster | Plaster | Gansu province 2018 | Musculo-skeletal system | 1 | NO |
| 270 | Fluphenazine Decanoate | Injection | Shanghai 2018 | Nervous system | 1 | NO |
| 271 | Ketelin | Capsule | Gansu province 2018 | Respiratory system | 1 | NO |
| 272 | Ketelin | Tablet | Gansu province 2018 | Respiratory system | 1 | NO |
| 273 | Reduced Glutathione | Eye Drops | Gansu province 2018 | Various | 1 | NO |
| 274 | Warfarin | Tablet | Hebei province 2018 | Blood and blood forming organs | 1 | NO |
| 275 | Ciclosporin | Tablet | Hunan province 2018 | Antineoplastic and immunomodulating agents | 1 | NO |
| 276 | Ciprofloxacin | Ear Drops | Gansu province 2018 | Sensory organs | 1 | NO |
| 277 | Ciprofloxacin | Cream | Gansu province 2018 | Dermatologicals | 1 | NO |
| 278 | Sulfadiazine Sodium | Injection | Hebei province 2018 | Anti-infectives for systemic use | 1 | NO |
| 279 | Menadiol | Tablet | Liaoning province 2020 | Blood and blood forming organs | 1 | NO |
| 280 | Medroxyprogesterone | Tablet | Gansu province 2018 | Genito urinary system and sex hormones | 1 | NO |
| 281 | Thiamazole | Tablet (enteric-coated) | Shanghai 2021 | Systematic hormonal preparations, sex hormones and insulins | 1 | NO |
| 282 | Methylrosanilinium Chloride | Topical solution | Hunan province 2018 | Dermatologicals | 1 | NO |
| 283 | Phloroglucinol | Injection | Hunan province 2020 | Alimentary tract and metabolism | 1 | NO |
| 284 | Defibrase | Injection | Gansu province 2018 | Blood and blood forming organs | 1 | NO |
| 285 | Purified Protein Derivative of Tuberculin (TB-PPD) | Injection | Shanghai 2019 | Various | 1 | NO |
| 286 | Polygeline | Injection | Shaanxi province 2019 | Blood and blood forming organs | 1 | NO |
| 287 | Carbachol | Injection | Inner Mongolia Autonomous Region 2018 | Sensory organs | 1 | NO |
| 288 | Carmustine | Injection | Tianjin 2021 | Antineoplastic and immunomodulating agents | 1 | NO |
| 289 | Glycerol Enema | Enema | Hebei province 2018 | Alimentary tract and metabolism | 1 | NO |
| 290 | Kanggongyan | Tablet | Gansu province 2018 | Genito urinary system and sex hormones | 1 | NO |
| 291 | Rabies Antiserum | Injection | Liaoning province 2020 | Anti-infectives for systemic use | 1 | NO |
| 292 | Agkistrodon Acutus Antivenin | Injection | Liaoning province 2020 | Anti-infectives for systemic use | 1 | NO |
| 293 | Bungarus Multicnctus Antivenin | Injection | Liaoning province 2020 | Anti-infectives for systemic use | 1 | NO |
| 294 | Tripterysium Glycosides | Tablet | Gansu province 2019 | Antineoplastic and immunomodulating agents | 1 | NO |
| 295 | Ribavirin | Tablet | Hunan province 2018 | Anti-infectives for systemic use | 1 | NO |
| 296 | Ribavirin | Injection | Hunan province 2018 | Anti-infectives for systemic use | 1 | NO |
| 297 | Rifandin | Capsule | Gansu province 2018 | Anti-infectives for systemic use | 1 | NO |
| 298 | Rifampicin | Tablet | Liaoning province 2021 | Anti-infectives for systemic use | 1 | NO |
| 299 | Ritodrine | Tablet | Shanxi Province 2020 | Genito urinary system and sex hormones | 1 | NO |
| 300 | Bifendate | Pill | Gansu province 2018 | Alimentary tract and metabolism | 1 | NO |
| 301 | Bifendate | Tablet | Gansu province 2018 | Alimentary tract and metabolism | 1 | NO |
| 302 | Streptomycin | Injection | Gansu province 2018 | Anti-infectives for systemic use | 1 | NO |
| 303 | Amphotericin B Liposome | Injection | Guangxi Zhuang Autonomous Region 2021 | Anti-infectives for systemic use | 1 | NO |
| 304 | Benproperine | Dilayer Sustained Release Tablets | Gansu province 2018 | Respiratory system | 1 | NO |
| 305 | Benproperine | Tablet (dispersible) | Gansu province 2018 | Respiratory system | 1 | NO |
| 306 | Benproperine | Oral liquid | Gansu province 2018 | Respiratory system | 1 | NO |
| 307 | Calcium Hydrogen Phosphate | Tablet | Tianjin 2021 | Alimentary tract and metabolism | 1 | NO |
| 308 | Barium Sulfate For Suspension（Type Ⅰ） | Suspension | Shanghai 2019 | Various | 1 | NO |
| 309 | Ferrous Sulfate | Sustained-release Tablet | Gansu province 2018 | Blood and blood forming organs | 1 | NO |
| 310 | Rotundin | Tablet | Guangxi Zhuang Autonomous Region 2019 | Nervous system | 1 | NO |
| 311 | Lodoxamide | Eye Drops | Gansu province 2018 | Sensory organs | 1 | NO |
| 312 | Lomustine | Capsule | Gansu province 2018 | Antineoplastic and immunomodulating agents | 1 | NO |
| 313 | Loperamide | Capsule | Gansu province 2018 | Alimentary tract and metabolism | 1 | NO |
| 314 | Chlorphenamine | Tablet | Hebei province 2018 | Respiratory system | 1 | NO |
| 315 | Clozapine | Tablet | Gansu province 2018 | Nervous system | 1 | NO |
| 316 | Calcium Chloride and Sodium Bromide | Injection | Hainan province 2021 | Nervous system | 1 | NO |
| 317 | Ephedrine | Nasal Drops | Guangxi Zhuang Autonomous Region 2019 | Respiratory system | 1 | NO |
| 318 | Ephedrine | Injection | Shanghai 2020 | Respiratory system | 1 | NO |
| 319 | Maren Pills | Pill | Gansu province 2018 | Alimentary tract and metabolism | 1 | NO |
| 320 | Mexiletine | Tablet | Jiangsu province 2021 | Cardiovascular system | 1 | NO |
| 321 | Sterile Water | Injection | Gansu province 2019 | Various | 1 | NO |
| 322 | Natamycin | Eye Drops | Liaoning province 2021 | Sensory organs | 1 | NO |
| 323 | Naphazoline | Nasal Drops | Gansu province 2018 | Respiratory system | 1 | NO |
| 324 | Nicardipine | Sustained-release Capsule | Hubei province 2021 | Cardiovascular system | 1 | NO |
| 325 | Nimodipine | Tablet | Yunnan province 2018 | Cardiovascular system | 1 | NO |
| 326 | Nimustine | Injection | Gansu province 2018 | Antineoplastic and immunomodulating agents | 1 | NO |
| 327 | Colistin | Injection | Guizhou province 2020 | Anti-infectives for systemic use | 1 | NO |
| 328 | Paroxetine | Tablet | Gansu province 2018 | Nervous system | 1 | NO |
| 329 | Prednisolone | Injection | Jiangsu province 2021 | Systematic hormonal preparations, sex hormones and insulins | 1 | NO |
| 330 | Primidone | Tablet | Gansu province 2018 | Nervous system | 1 | NO |
| 331 | Sodium Glucuronic Acid | Injection | Gansu province 2018 | Alimentary tract and metabolism | 1 | NO |
| 332 | Sodium Stibogluconate | Injection | Liaoning province 2020 | Antiparasitic products, insecticides and repellents | 1 | NO |
| 333 | Prolonium Iodide | Injection | Shanghai 2019 | Sensory organs | 1 | NO |
| 334 | Ziprasidone | Injection | Gansu province 2018 | Nervous system | 1 | NO |
| 335 | Hydroxocobalamin | Injection | Liaoning province 2021 | Blood and blood forming organs | 1 | NO |
| 336 | Hydroxycarbamide | Capsule | Hubei province 2021 | Antineoplastic and immunomodulating agents | 1 | NO |
| 337 | Sodium Chloride Hydroxyethyl Starch 40 | Injection | Guangxi Zhuang Autonomous Region 2019 | Blood and blood forming organs | 1 | NO |
| 338 | Penicillamine | Injection | Shandong province 2021 | Musculo-skeletal system | 1 | NO |
| 339 | Benzylpenicillin Sodium | Injection | Hainan province 2021 | Anti-infectives for systemic use | 1 | NO |
| 340 | Hydrochlorothiazide | Tablet | Gansu province 2018 | Cardiovascular system | 1 | NO |
| 341 | Gentamycin | Eye Drops | Gansu province 2018 | Sensory organs | 1 | NO |
| 342 | Colchicine | Tablet | Ningxia Hui Autonomous Region 2020 | Musculo-skeletal system | 1 | NO |
| 343 | Deferiprone | Tablet | Guangdong province 2019 | Various | 1 | NO |
| 344 | Ethinylestradiol and Cyproterone Acetate | Tablet | Inner Mongolia Autonomous Region 2018 | Genito urinary system and sex hormones | 1 | NO |
| 345 | Cytidine Disodium Triphosphate | Injection | Gansu province 2018 | Nervous system | 1 | NO |
| 346 | Adenosine Disodium Triphosphate | Tablet | Hunan province 2018 | Various | 1 | NO |
| 347 | Adenosine Disodium Triphosphate | Injection | Jiangsu province 2021 | Various | 1 | NO |
| 348 | Sanqi | Capsule | Gansu province 2018 | Blood and blood forming organs | 1 | NO |
| 349 | Sanqi | Tablet | Gansu province 2018 | Blood and blood forming organs | 1 | NO |
| 350 | Thalidomide | Capsule | Gansu province 2018 | Antineoplastic and immunomodulating agents | 1 | NO |
| 351 | Batilol | Tablet | Inner Mongolia Autonomous Region 2018 | Blood and blood forming organs | 1 | NO |
| 352 | Anisodamine | Injection | Guangxi Zhuang Autonomous Region 2018 | Alimentary tract and metabolism | 1 | NO |
| 353 | Damp-removing-Pain-killing Ointment | Cream | Gansu province 2018 | Musculo-skeletal system | 1 | NO |
| 354 | Testosterone Undecanoate | Injection | Gansu province 2019 | Genito urinary system and sex hormones | 1 | NO |
| 355 | Sulpiride | Tablet | Gansu province 2018 | Nervous system | 1 | NO |
| 356 | Instant-effect Jiuxin Pills | Pill | Hunan province 2020 | Cardiovascular system | 1 | NO |
| 357 | Sodium Bicarbonate | Oral powder | Guangdong province 2019 | Alimentary tract and metabolism | 1 | NO |
| 358 | Teniposide | Injection | Hebei province 2018 | Antineoplastic and immunomodulating agents | 1 | NO |
| 359 | Oxytetracycline | Tablet | Gansu province 2018 | Anti-infectives for systemic use | 1 | NO |
| 360 | Tobramycin | Injection | Shanxi Province 2020 | Anti-infectives for systemic use | 1 | NO |
| 361 | Thiamine | Tablet | Gansu province 2018 | Alimentary tract and metabolism | 1 | NO |
| 362 | Vitamin B | Injection | Gansu province 2018 | Alimentary tract and metabolism | 1 | NO |
| 363 | Cyanocobalamin | Tablet | Gansu province 2018 | Blood and blood forming organs | 1 | NO |
| 364 | Pyridoxine | Tablet | Yunnan province 2018 | Alimentary tract and metabolism | 1 | NO |
| 365 | Colecalciferol | Injection | Yunnan province 2018 | Alimentary tract and metabolism | 1 | NO |
| 366 | Estradiol valerate | Tablet | Inner Mongolia Autonomous Region 2018 | Genito urinary system and sex hormones | 1 | NO |
| 367 | Complex Packing Estradiol Valerate Tablets, Estradiol Valerate And Cyproterone Acetate Tablets | Tablet | Yunnan province 2018 | Genito urinary system and sex hormones | 1 | NO |
| 368 | Cimetidine | Tablet | Inner Mongolia Autonomous Region 2018 | Alimentary tract and metabolism | 1 | NO |
| 369 | Cimetidine | Injection | Guangxi Zhuang Autonomous Region 2021 | Alimentary tract and metabolism | 1 | NO |
| 370 | Absorbable Gelatin Sponge | Sponge | Shanghai 2020 | Blood and blood forming organs | 1 | NO |
| 371 | Erosion-removal suppository | Suppository | Gansu province 2018 | Genito urinary system and sex hormones | 1 | NO |
| 372 | Nifedipine | Sustained-release Tablet | Yunnan province 2018 | Cardiovascular system | 1 | NO |
| 373 | Nitroglycerin | Spray | Jilin province 2020 | Cardiovascular system | 1 | NO |
| 374 | Pilocarpine | Eye Drops | Inner Mongolia Autonomous Region 2018 | Nervous system | 1 | NO |
| 375 | Clonazepam | Tablet | Hunan province 2018 | Nervous system | 1 | NO |
| 376 | Minor Bupleurum | Granule | Yunnan province 2018 | Nervous system | 1 | NO |
| 377 |  | Granule | Gansu province 2018 | Respiratory system | 1 | NO |
| 378 | Pediatric digestion tablets | Tablet | Gansu province 2018 | Alimentary tract and metabolism | 1 | NO |
| 379 | Actinomycin | Injection | Hunan province 2018 | Antineoplastic and immunomodulating agents | 1 | NO |
| 380 | Ursodeoxycholic Acid | Tablet | Yunnan province 2018 | Alimentary tract and metabolism | 1 | NO |
| 381 | Hematoporphyrin | Injection | Gansu province 2018 | Antineoplastic and immunomodulating agents | 1 | NO |
| 382 | Nicotinic Acid | Tablet | Gansu province 2018 | Cardiovascular system | 1 | NO |
| 383 | Nicotinic Acid | Injection | Shanghai 2021 | Cardiovascular system | 1 | NO |
| 384 | Berberine | Tablet | Yunnan province 2018 | Alimentary tract and metabolism | 1 | NO |
| 385 | Ocular Extractives | Injection | Gansu province 2018 | Sensory organs | 1 | NO |
| 386 | Yangyin Qingfei pill | Pill | Gansu province 2018 | Respiratory system | 1 | NO |
| 387 | Sodium calcium edetate | Cream | Gansu province 2018 | Various | 1 | NO |
| 388 | Sodium calcium edetate | Injection | Liaoning province 2021 | Various | 1 | NO |
| 389 | Insulin | Injection | Ningxia Hui Autonomous Region 2018 | Alimentary tract and metabolism | 1 | NO |
| 390 | Ethambutol | Tablet | Hebei province 2018 | Anti-infectives for systemic use | 1 | NO |
| 391 | Pyrimethamine | Tablet | Liaoning province 2020 | Antiparasitic products, insecticides and repellents | 1 | NO |
| 392 | Acetazolamide | Tablet | Liaoning province 2020 | Sensory organs | 1 | NO |
| 393 | Human Hepatitis B Immunoglobulin (Ph4) | Injection | Shaanxi province 2019 | Anti-infectives for systemic use | 1 | NO |
| 394 | Isosorbitol | Oral liquid | Tianjin 2021 | Cardiovascular system | 1 | NO |
| 395 | Fructus forsythiae antidotal tablets | Tablet | Yunnan province 2018 | Respiratory system | 1 | NO |
| 396 | Indometacin | Tablet | Gansu province 2018 | Musculo-skeletal system | 1 | NO |
| 397 | Fluorescein Sodium | Injection | Gansu province 2018 | Sensory organs | 1 | NO |
| 398 | Iron Dextran | Injection | Gansu province 2018 | Alimentary tract and metabolism | 1 | NO |
| 399 | Yunnan pai yao | Capsule | Yunnan province 2018 | Musculo-skeletal system | 1 | NO |
| 400 | Zhenju Jiangya Tablet | Tablet | Gansu province 2018 | Cardiovascular system | 1 | NO |
| 401 | Recombinant Human Interferon Α2B | Eye Drops | Shaanxi province 2020 | Sensory organs | 1 | NO |
| 402 | Recombinant Human Interferon Α2B Gel | Vaginal Gel | Hunan province 2018 | Dermatologicals | 1 | NO |
| 403 | Recombinant Human Insulin | Injection | Gansu province 2018 | Alimentary tract and metabolism | 1 | NO |
| 404 | Inhalatio Amylis | Inhalation | Heilongjiang province 2020 | Various | 1 | NO |
| 405 | Sulodexide | Injection | Heilongjiang province 2020 | Blood and blood forming organs | 1 | NO |
| 406 | Jidesheng Sheyao Tablet | Tablet | Hubei province 2021 | Various | 1 | NO |
| 407 | Qinglong Sheyao Tablet | Tablet | ^Hubei^ province 2021 | Various | 1 | NO |
| 408 | Shanghai Sheyao Table | Tablet | Hubei province 2021 | Various | 1 | NO |

**Table S2.**  Drug shortages in both NEML and WHO Model List in China, 2018-2021.

| No. | Name | Dosage form | Therapeutic category | Frequency in the provincial shortage lists | Whether in the national list of drug shortages |
| --- | --- | --- | --- | --- | --- |
|  |  |  |  |  |  |
| 1 | Methotrexate | Injection | Antineoplastic and immunomodulating agents | 26 | YES |
| 2 | Protamine | Injection | Various | 21 | YES |
| 3 | Atropine | Injection | Alimentary tract and metabolism | 20 | YES |
| 4 | Neostigmine | Injection | Nervous system | 17 | YES |
| 5 | Pyridostigmine Bromide | Tablet | Nervous system | 17 | YES |
| 6 | Nitroglycerin | Tablet | Genito urinary system and sex hormones | 17 | YES |
| 7 | Cytarabine | Injection | Antineoplastic and immunomodulating agents | 15 | YES |
| 8 | Allopurinol | Tablet | Musculo-skeletal system | 15 | YES |
| 9 | Calcium gluconate | Injection | Alimentary tract and metabolism | 15 | YES |
| 10 | Adrenaline | Injection | Cardiovascular system | 15 | YES |
| 11 | Benzathine Benzylpenicillin | Injection | Anti-infectives for systemic use | 14 | YES |
| 12 | Ketamine | Injection | Nervous system | 13 | NO |
| 13 | Phenobarbital | Injection | Nervous system | 12 | YES |
| 14 | Sodium Thiosulfate | Injection | Various | 12 | YES |
| 15 | Oxytocin | Injection | Systematic hormonal preparations, sex hormones and insulins | 12 | YES |
| 16 | Verapamil | Injection | Cardiovascular system | 12 | YES |
| 17 | Diazepam | Injection | Nervous system | 11 | YES |
| 18 | Furosemide | Injection | Cardiovascular system | 11 | YES |
| 19 | Magnesium Sulfate | Injection | Nervous system | 11 | YES |
| 20 | Vincristine | Injection | Antineoplastic and immunomodulating agents | 11 | YES |
| 21 | Hydroxycarbamide | Tablet | Antineoplastic and immunomodulating agents | 10 | NO |
| 22 | Cyclophosphamide | Injection | Antineoplastic and immunomodulating agents | 9 | YES |
| 23 | Methylene Blue | Injection | Various | 9 | YES |
| 24 | Chlorpromazine | Injection | Nervous system | 9 | NO |
| 25 | Hydrocortisone | Injection | Systematic hormonal preparations, sex hormones and insulins | 8 | YES |
| 26 | Mercaptopurine | Tablet | Antineoplastic and immunomodulating agents | 8 | YES |
| 27 | Penicillamine | Tablet | Musculo-skeletal system | 7 | YES |
| 28 | Amiodarone | Injection | Cardiovascular system | 7 | YES |
| 29 | Digoxin | Oral liquid | Cardiovascular system | 7 | YES |
| 30 | Etoposide | Injection | Antineoplastic and immunomodulating agents | 7 | YES |
| 31 | Haloperidol | Injection | Nervous system | 7 | NO |
| 32 | Heparin | Injection | Blood and blood forming organs | 7 | NO |
| 33 | Sodium Nitroprusside | Injection | Cardiovascular system | 6 | YES |
| 34 | Bupivacaine | Injection | Nervous system | 6 | NO |
| 35 | Propranolol | Tablet | Cardiovascular system | 6 | NO |
| 36 | Metoclopramide | Injection | Alimentary tract and metabolism | 6 | NO |
| 37 | Testosterone Propionate | Injection | Genito urinary system and sex hormones | 5 | NO |
| 38 | Metoclopramide | Tablet | Alimentary tract and metabolism | 5 | NO |
| 39 | Folic acid | Tablet | Blood and blood forming organs | 5 | NO |
| 40 | Naloxone | Injection | Nervous system | 4 | YES |
| 41 | Metronidazole | Tablet | Antiparasitic products, insecticides and repellents | 4 | NO |
| 42 | Tamoxifen | Tablet | Antineoplastic and immunomodulating agents | 4 | NO |
| 43 | Verapamil | Tablet | Cardiovascular system | 4 | NO |
| 44 | Mannitol | Injection | Blood and blood forming organs | 3 | YES |
| 45 | Praziquantel | Tablet | Antiparasitic products, insecticides and repellents | 3 | NO |
| 46 | Digoxin | Tablet | Cardiovascular system | 3 | NO |
| 47 | Dexamethasone | Tablet | Systematic hormonal preparations, sex hormones and insulins | 3 | NO |
| 48 | Fluorouracil | Injection | Antineoplastic and immunomodulating agents | 3 | NO |
| 49 | Tropicamide | Eye Drops | Sensory organs | 3 | NO |
| 50 | Erythromycin | Eye ointment | Sensory organs | 3 | NO |
| 51 | Carbamazepine | Tablet | Nervous system | 3 | NO |
| 52 | Amphotericin B | Injection | Anti-infectives for systemic use | 3 | NO |
| 53 | Fosfomycin | Injection | Anti-infectives for systemic use | 3 | NO |
| 54 | Human Tetanus Immunoglobulin | Injection | Anti-infectives for systemic use | 3 | NO |
| 55 | (Human) Coagulation Factor Ⅷ | Injection | Blood and blood forming organs | 3 | NO |
| 56 | Erythromycin lactobionate | Injection | Anti-infectives for systemic use | 3 | NO |
| 57 | Lithium Carbonate | Tablet | Nervous system | 3 | NO |
| 58 | Ifosfamide | Injection | Antineoplastic and immunomodulating agents | 3 | NO |
| 59 | Tranexamic acid | Injection | Blood and blood forming organs | 2 | NO |
| 60 | Phenobarbital Sodium | Tablet | Nervous system | 2 | NO |
| 61 | Sodium Valproate | Tablet | Nervous system | 2 | NO |
| 62 | Lidocaine | Injection | Cardiovascular system | 2 | NO |
| 63 | Azathioprine | Tablet | Antineoplastic and immunomodulating agents | 2 | NO |
| 64 | Calamine | Lotion | Dermatologicals | 2 | NO |
| 65 | Chlorpromazine | Tablet | Nervous system | 2 | NO |
| 66 | Hydrocortisone | Tablet | Systematic hormonal preparations, sex hormones and insulins | 2 | NO |
| 67 | Vitamin B | Injection | Alimentary tract and metabolism | 2 | NO |
| 68 | Nifedipine | Tablet | Cardiovascular system | 2 | NO |
| 69 | Isosorbide Dinitrate | Tablet | Cardiovascular system | 2 | NO |
| 70 | Calcium Folinate | Injection | Various | 2 | NO |
| 71 | Isoniazid | Tablet | Anti-infectives for systemic use | 2 | NO |
| 72 | Rifampicin | Capsule | Anti-infectives for systemic use | 2 | NO |
| 73 | Albendazole | Tablet | Antiparasitic products, insecticides and repellents | 1 | NO |
| 74 | Amikacin | Injection | Anti-infectives for systemic use | 1 | NO |
| 75 | Aciclovir | Tablet | Anti-infectives for systemic use | 1 | NO |
| 76 | Phenytoin Sodium | Injection | Nervous system | 1 | NO |
| 77 | Pyrazinamide | Tablet | Anti-infectives for systemic use | 1 | NO |
| 78 | Primaquine | Tablet | Antiparasitic products, insecticides and repellents | 1 | NO |
| 79 | Budesonide | inhalation | Respiratory system | 1 | NO |
| 80 | Dexamethasone Acetate | Tablet | Systematic hormonal preparations, sex hormones and insulins | 1 | NO |
| 81 | Digoxin | Injection | Cardiovascular system | 1 | NO |
| 82 | Diazepam | Tablet | Nervous system | 1 | NO |
| 83 | Furosemide | Tablet | Cardiovascular system | 1 | NO |
| 84 | Haloperidol | Tablet | Nervous system | 1 | NO |
| 85 | Warfarin | Tablet | Blood and blood forming organs | 1 | NO |
| 86 | Medroxyprogesterone | Tablet | Genito urinary system and sex hormones | 1 | NO |
| 87 | Purified Protein Derivative of Tuberculin (TB-PPD) | Injection | Various | 1 | NO |
| 88 | Ribavirin | Tablet | Anti-infectives for systemic use | 1 | NO |
| 89 | Rifampicin | Tablet | Anti-infectives for systemic use | 1 | NO |
| 90 | Streptomycin | Injection | Anti-infectives for systemic use | 1 | NO |
| 91 | Ferrous Sulfate | Sustained-release Tablet | Blood and blood forming organs | 1 | NO |
| 92 | Loperamide | Capsule | Alimentary tract and metabolism | 1 | NO |
| 93 | Clozapine | Tablet | Nervous system | 1 | NO |
| 94 | Sodium Stibogluconate | Injection | Antiparasitic products, insecticides and repellents | 1 | NO |
| 95 | Benzylpenicillin Sodium | Injection | Anti-infectives for systemic use | 1 | NO |
| 96 | Hydrochlorothiazide | Tablet | Cardiovascular system | 1 | NO |
| 97 | Testosterone Undecanoate | Injection | Genito urinary system and sex hormones | 1 | NO |
| 98 | Pyridoxine | Tablet | Alimentary tract and metabolism | 1 | NO |
| 99 | Pilocarpine | Eye Drops | Nervous system | 1 | NO |
| 100 | Insulin | Injection | Alimentary tract and metabolism | 1 | NO |
| 101 | Ethambutol | Tablet | Anti-infectives for systemic use | 1 | NO |
| 102 | Pyrimethamine | Tablet | Antiparasitic products, insecticides and repellents | 1 | NO |
| 103 | Acetazolamide | Tablet | Sensory organs | 1 | NO |
| 104 | Recombinant Human Insulin | Injection | Alimentary tract and metabolism | 1 | NO |

NEML, National Essential Medicine List; WHO Model List, World Health Organization Model List of Essential Medicines

**Table S3.** Drug shortages in China on each list, 2018-2021.

| Attribute types | | Number of varieties | Proportion/% | ratio |
| --- | --- | --- | --- | --- |
| NEML |  |  |  |  |
|  | Essential medicine | 208 | 51.0% | 208/685 |
|  | Non-essential medicine | 200 | 49.0% | — |
| WHO Model List of Essential Medicines |  |  |  |  |
|  | Essential medicine | 131 | 32.1% | 131/479 |
|  | Non-essential medicine | 277 | 67.9% | — |
| NMIDL |  |  |  |  |
|  | Medical insurance category A | 224 | 54.9% | 224/641 |
|  | Medical insurance category B | 110 | 27.0% | 110/2219 |
|  | Non-medical insurance drug | 74 | 18.1% | — |
| VBPL |  |  |  |  |
|  | volume-based purchasing drug | 14 | 3.4% | 14/236 |
|  | Non-volume-based purchasing drug | 394 | 96.6% | — |

NMIDL, National Medical Insurance Drug List; VBPL, Volume-Based Purchasing List

**Figure S1.** Frequency distribution of drug shortages in the provincial lists of drug shortages in China, 2018-2021.
